# Structural variations in evolutionary novel genomic regions: new insights into neurodevelopmental disorders by long-read DNA Sequencing

**DOI:** 10.1186/s10020-025-01415-y

**Published:** 2026-01-09

**Authors:** Martina Rincic, Janja Kopic, Valentina Klein, Zeljka Krsnik, Thomas Liehr, Sebastian Giesselmann, Ingo Kurth, Florian Kraft

**Affiliations:** 1 Croatian Institute for Brain Research, School of Medicine, University of Zagreb, Zagreb, 10000 Croatia; 2 Jena University Hospital, Friedrich Schiller University, Institute of Human Genetics, Jena, Germany; 3 Center for Human Genetics and Genomic Medicine, Faculty of Medicine, RWTH Aachen University, Aachen, Germany

**Keywords:** Oxford Nanopore long-read DNA sequencing, Neurodevelopmental disorders, *NBPF* and *RGPD* gene family, *ANKRD20A1*, Human-specific genomic regions, FISH, Immunofluorescence, Developing human cortex

## Abstract

**Background:**

Neurodevelopmental disorders (NDDs) are highly diverse conditions often associated with genetic abnormalities. However, a large number of NDD cases remain undiagnosed despite thorough genetic testing using short-read sequencing and chromosomal microarray analysis. Emerging evidence indicates that structural variants (SVs) in evolutionarily new and human-specific genomic regions may be responsible for these unresolved cases.

**Methods:**

We used Oxford Nanopore long-read DNA sequencing in six patients with unexplained NDDs who had previously tested negative for genetic mutations. Structural variants were identified and filtered based on their genomic location, regulatory potential, and expression in the central nervous system. Confirmatory fluorescence in situ hybridization (FISH) was performed. Additionally, immunohistochemical analysis of the candidate gene *ANKRD20A1* was carried out to assess its spatiotemporal expression in the developing human brain. Interactome analysis was also performed to evaluate the functional connections of genes impacted by SVs.

**Results:**

A total of twenty-six candidate SVs were found, including deletions, duplications, insertions, and inversions. Many of these SVs affect rapidly evolving gene families such as NBPF, TBC1D3, and RGPD, as well as regulatory elements in brain-expressed genes, suggesting their potential to disrupt brain development and lead to NDD. FISH analysis confirmed several large SVs. Patient-specific interactome maps showed that most RGPD-disrupted genes create extensive interactions. Immunohistochemical analysis revealed that *ANKRD20A1*, a candidate gene, is dynamically expressed during midfetal human cortical development, suggesting its involvement in neurodevelopmental events.

**Conclusion:**

Our findings highlight the crucial role of long-read DNA sequencing in uncovering concealed structural variants within recently evolved genomic regions. These SVs may contribute to the development of NDDs by disrupting coding sequences, regulatory elements, or complex gene networks. This study supports integrating long-read sequencing into research workflows.

**Supplementary Information:**

The online version contains supplementary material available at 10.1186/s10020-025-01415-y.

## Background

Neurodevelopmental disorders (NDDs) encompass a diverse spectrum of conditions characterized by impairments in the growth and development of the nervous system. NDDs typically manifest early in childhood and persist throughout one’s lifespan, significantly impacting cognitive, emotional, and behavioral functioning. NDDs affect a substantial proportion of the global population, with varying degrees of severity and complexity. According to recent epidemiological studies, the prevalence of NDDs fluctuates globally between 5 and 20% worldwide, making them among the most prevalent categories of childhood-onset disabilities (Francés et al. 2022). The genetic bases of NDDs are well established. However, despite the growing use of genome and exome sequencing (GS/ES), chromosomal microarray (CMA), and optical mapping to identify molecular diagnoses for rare diseases, diagnostic success rates vary widely, ranging from as low as 20% to as high as 60% (Clark et al. 2018). This suggests that suspected genetic conditions remain unresolved through current genomic testing methods. This could be attributed to several reasons. While some cases may involve phenotypes influenced by polygenic or environmental factors (Vears et al. 2018), other undiagnosed cases likely originate from genetic variants that current genomic technologies fail to detect. Short-read/next-generation sequencing (NGS), for example, has limited sensitivity to certain types of variants, particularly structural variants (SVs) and those affecting repetitive sequences, but is highly sensitive to single-nucleotide variants (SNVs) and small indels in gene-rich regions. CMA is a powerful tool for detecting chromosomal abnormalities, such as copy number variations (CNVs), but it has limitations, including its scope and resolution. In contrast to NGS and CMA, long-read/third-generation sequencing has been shown to enhance the detection of these challenging variants and enable more accurate structural variation analysis through de novo genome assemblies. As a result, long-read sequencing holds significant promise for advancing the diagnosis of rare diseases (Logsdon et al. 2020). It has already started to take place in the diagnosis of a wide range of diseases. This is especially emphasized in the diagnostic workup of patients diagnosed with NDDs with no previously identified pathogenic variants from NGS or CMA (Wenger et al. 2019; Hiatt et al. 2024).

NDDs are not restricted to the human species. Many genes and pathways implicated in human neurodevelopmental processes and linked to NDDs are conserved across species. For example, the *Mecp2* and *Fmr1* knockout mouse strains are used as models of human Rett syndrome and Fragile X syndrome, respectively, as they exhibit neurological and behavioral deficits similar to those observed in human conditions (Begley et al. 2012; Baldarelli et al. 2021; Baldarelli et al. 2024). Furthermore, animals with various genetic alterations can exhibit difficulties in social interaction, repetitive behaviors, or cognitive challenges analogous to autism spectrum disorder (ASD) and other neurodevelopmental conditions (Wilson et al. 2024). While the underlying genetic mechanisms and some features of NDDs are shared across species, the full scope and expression of NDDs, especially those involving greater cognitive and social functions, are unique primarily to humans. Higher cognitive functions are among the defining characteristics of modern humans, distinguishing them from all other species. This, together with the fact that NDDs have a strong genetic component, raises an intriguing question: Are there genetic traits unique to humans that contributed to the evolution of our brain development and function, and could these traits help explain the lack of molecular diagnoses for some rare diseases?

Genetic differences between species may result from distinct mechanisms, such as changes in genomic alterations, e.g., local chromosomal rearrangements, gene (family) duplications, and SNVs, causing differences in gene expression and alternative splicing (Fair and Pollen 2023; Whalen et al. 2023; Phillips 2021; Rodrigues et al. 2024). The functional consequences of these genetic changes can be observed in general behavior, tissue and organ development and function, as well as in molecular circuits, pathways, and cellular variations (Schmidt and Polleux 2022). Owing to the structure of these novel genomic regions, which are primarily composed of segmental duplications (Bibber et al. 2020), technical limitations have left us partially blind to their full complexity (Pollen et al. 2023). As a result, fully exploring the complete spectrum of SVs within these evolutionarily novel genomic regions has been impossible. However, long-read sequencing enables more comprehensive and accurate read alignment, particularly in these highly repetitive and rapidly evolving genome regions (Mitsuhashi and Matsumoto 2020; Ziaei Jam et al. 2024).

We utilized Nanopore long-read sequencing in seven patients with NDDs and associated phenotypic abnormalities. All patients had previously undergone genetic testing, including karyotyping, multiplex ligation-dependent probe amplification (MLPA), CMA, and exome NGS; however, no causal or potentially causal genetic variants were identified. Based on available literature data, long-read sequencing analysis has focused on human-specific genomic regions (Shao et al. 2019). Next, we used immunofluorescence to examine the expression patterns of the selected candidate gene in the developing human frontal cortex throughout the midfetal period, to establish its possible involvement in the cognitive phenotype of intellectual disability.

## Methods

### Sample selection and experimental procedure

The study included individuals diagnosed with neurodevelopmental disorders, including intellectual disability, together with epilepsy, underdeveloped speech, and associated clinical features, who had previously undergone genetic screening by karyotyping, CMA, and exome sequencing, with no causal genetic variation or only a VUS was identified, except for ST-4. Informed consent was obtained from parents or legal representatives for all patients participating in the study.

### Exome sequencing

Library preparation of the DNA samples from the patients was carried out using the IDT xGen™ DNA EZ UNI Library Prep Kit, IDT xGen Hybridization and Wash, and IDT xGen Exome Research Panel (v2.0) according to the manufacturer's protocol. The libraries were sequenced on a NovaSeq 6000 Sequencer with 2 × 159 cycles (mean coverage ~ 130x). Basecalling and secondary analysis was done using BCL convert (v4.2.4) and Illumina Dragen germline pipeline (v4.0), respectively. Annotation and bioinformatic prioritization of variants were carried out using KGGSeq (v1.1, 07/Feb./2019) together with an in-house bioinformatic pipeline. Variants with a minor allele frequency > 0.75% in public databases (i.e., gnomAD) and synonymous variants were not considered. CNVkit (v0.9.10) in combination with CNVizard was used for CNV calling (Krause et al. 2024).

### Long-read genome sequencing

The patients' DNA was sheared using the Megaruptor3 (Diagenode), and small fragments were depleted using 0.4 × bead clean-up (CleanNA CleanNGS beads) and a short-read elimination kit (PacBio). The libraries were prepared with the SQK-NBD114.24 kit (Oxford Nanopore Technologies) according to the manufacturer’s protocol, with 1–1.5 µg of size-selected DNA used as input. Sequencing was performed on a PromethION 24 sequencer (Oxford Nanopore Technologies) for 72 h, with nuclease flushes and reloads after 24 and 48 h, respectively. Base calling and data analysis were performed via Dorado (version 0.5) and the wf-human-variation pipeline (version 1.9.2). AnnotSV was used for annotation, and IGV was used for visualization of the SV data (Geoffroy et al. 2023).

### Fluorescence in situ hybridization (FISH) analysis

Following standard protocol, fluorescence in situ hybridization (FISH) with locus-specific bacterial artificial chromosome (BAC) probes was used to validate SVs detected via long-read DNA sequencing.

### Data analysis for SV prioritization

**Fig. 1:**
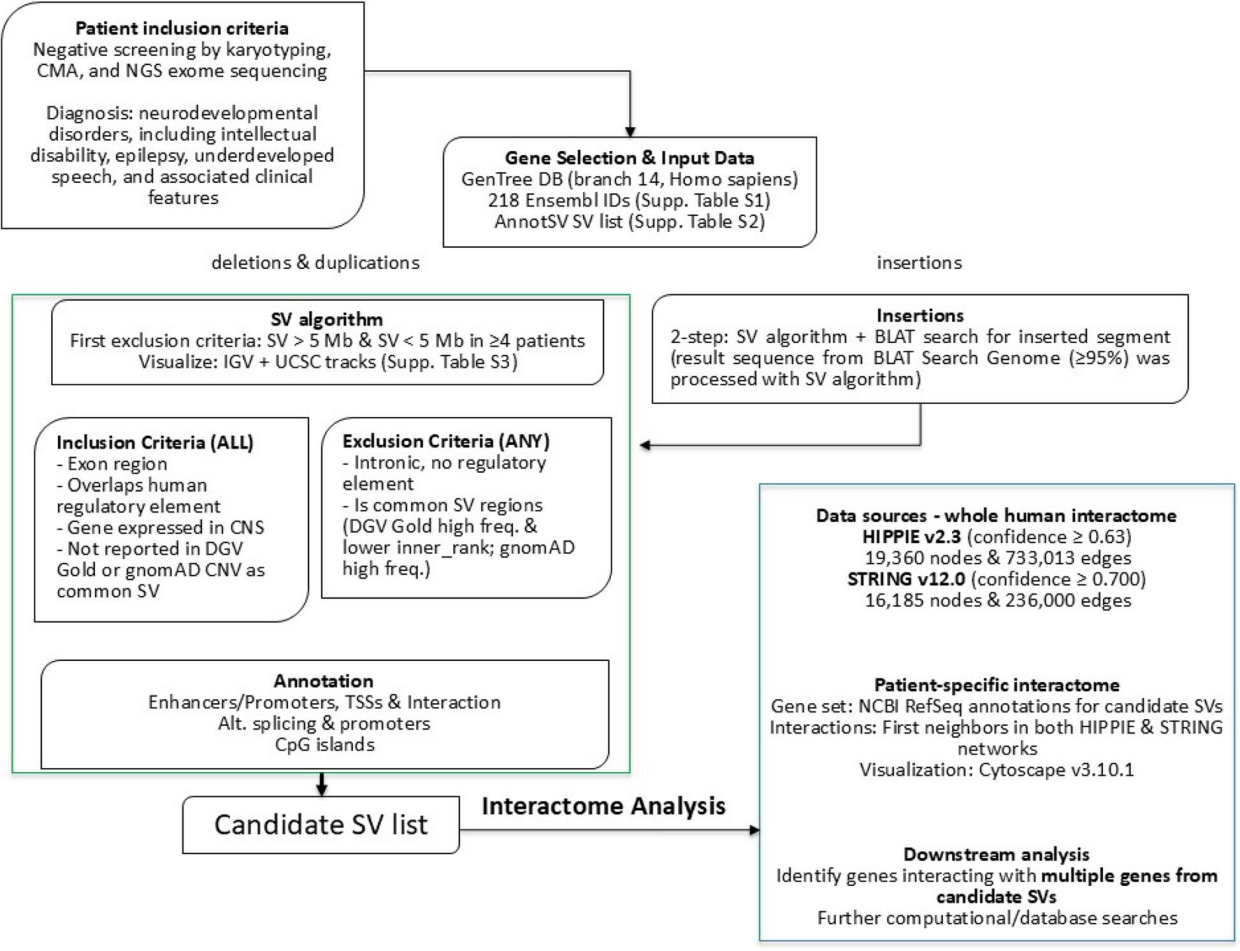
SV prioritization/interactome pipeline. Summary of the integrated analysis pipeline, including long-read sequencing (LRS), variant filtering, structural variant (SV) prioritization, and fluorescence in situ hybridization (FISH) validation, followed by interactome analysis

A schematic representation of the SV prioritization/interactome pipeline is shown in Fig. 1. Based on the literature data of the GenTree database, a set of genes was selected for analysis (Shao et al. 2019). In total, 218 Ensembl IDs for Homo sapiens (GRCh38/hg38) branch 14 were used for annotation (Supplemental Table S1). A complete list of detected SVs in all samples from AnnotSV is available in Supplemental Table S2. According to the results of genetic screening before sequencing, all SVs larger than 5 Mb were excluded from further analysis. Furthermore, all SVs smaller than 5 Mb observed in four or more patients were excluded from further analysis. The remaining SVs were visually inspected in IGV and displayed in UCSC using a specific set of tracks (Supplemental Table S3), and were further filtered based on several criteria. Exclusion criteria for SVs were that SV lies in the known region of normal structural variation in the general population. This was validated from the DGV Gold Standard and the gnomAD CNV database. From DGV Gold Standard databases, frequency, inner rank, and number of samples included were evaluated, and all SVs with high frequency and higher inner rank were excluded from further analysis. Variants with a lower inner rank in the DGV represent the highest breakpoint accuracy within their cluster and define the high-confidence (‘inner’) boundaries of the gold-standard variant, making them more reliable for precise genomic interpretation. On the other hand, higher inner ranks in the DGV indicate progressively lower-ranked supporting variants within that merged group. Briefly, a lower inner rank indicates a more reliably detected SV, while a higher rank indicates less reliability. Additionally, Population allele frequencies from the gnomAD CNV database were used to distinguish common, likely benign structural variants from rare CNVs of potential clinical relevance, providing essential context for result interpretation, and all SVs that had high frequency were excluded from further analysis. The following exclusion criterion was that the SV was located in an intronic region and that no regulatory elements were present. The inclusion criteria for further analysis were that the SV is located in the exon region, covers any human regulatory elements, and covers genes expressed in the central nervous system. The GeneHancer track in UCSC was applied to assess regulatory elements in SVs. GeneHancer is a database of human regulatory elements, including enhancers and promoters and their inferred target genes, integrated within GeneCards, a comprehensive human gene compendium. The GeneHancer track set includes regulatory elements (GeneHancers), gene transcription start sites (TSSs), interactions (associations) between regulatory elements and genes, and clustered interactions by gene target or GeneHancer. Additionally, alternative splicing, alternative promoters, and similar events in UCSC genes were examined. In the case of insertions, the BLAT Search Genome tool from UCSC was used to identify sequences with a similarity of 95% or greater, and a two-step approach was employed. First, the insertion position was evaluated using the algorithm described above for SV deletions and duplications; second, the BLAT result was used in conjunction with this algorithm. This resulted in the generation of the candidate SV list.

### Interactome analysis

Interactome analysis was performed to evaluate the simultaneous contribution of specific gene combinations from different SVs in a single patient. First, the whole human interactome was generated using the Human Integrated Protein–Protein Interaction Reference (HIPPIE) database (version 2.3) (Alanis-Lobato et al. 2017). Interactions with a confidence value below 0.63 (medium confidence) were filtered out. As a result, a human interactome comprising 19,360 nodes (genes) and 733,013 edges (interactions) was constructed. Next, the STRING interactome was downloaded, including AB pairs with a high confidence level of 0.700 (Version: 12.0). This resulted in an interactome containing 16,185 nodes (proteins) and 236,000 edges (interactions). NCBI RefSeq annotations were retrieved for candidate SVs in each patient to generate a patient-specific interactome. Protein‒Protein interactions were retrieved based on their first neighbors in both networks. Cytoscape 3.10.1 was used for interactome visualization (Shannon et al. 2003). Further computational and database analyses were performed to identify genes that interact with multiple genes from candidate SVs. A schematic representation of the SV prioritization/interactome pipeline is shown in Fig. 1.

### Histological and immunofluorescence analysis

The study was conducted using postmortem human fetal brain tissue from specimens aged 12–21 postconception weeks (PCW). All tissue collection procedures complied with the ethical guidelines outlined in the 2000 Declaration of Helsinki. The samples were obtained from the Zagreb Neuroembryological Collection, with ethical clearance granted by the Internal Review Board of the Ethical Committee at the University of Zagreb, School of Medicine. Additional brain material was provided through the Human Developmental Biology Resource, supported by Joint MRC/Wellcome Trust grant #099175/Z/12/Z. Following extraction, the fetal brains were preserved in a 4% paraformaldehyde (PFA) solution in 0.1 M phosphate-buffered saline (PBS) at pH 7.4. The gestational age of the fetuses was estimated based on crown–rump length (CRL) measurements and pregnancy documentation reported in PCWs. For analysis, the tissue was embedded in paraffin and then sectioned coronally into 10 μm slices by a Leica SM2000R microtome (Wetzlar, Germany).

The tissue sections were stained with classical Cresyl violet (Nissl) to visualize their cytoarchitecture. Immunofluorescence staining was performed according to our established laboratory protocol. In brief, the slides were first deparaffinized with xylene and rehydrated through a graded ethanol series (100% to 70% ethanol). For antigen retrieval, the sections were heat-treated in citrate buffer at pH 6.0. A blocking solution composed of 1% BSA and 0.5% Triton X-100 in 1 × PBS was applied. Primary antibodies (ANKRD20A1- polyclonal rabbit, LS-C805555, LsBio, 1:100; CTIP2- rat monoclonal, ab18465, Abcam, 1:500) were incubated overnight at 4 °C in a humidified chamber. The next day, the slides were washed in PBS and then incubated with secondary antibodies (AF488 donkey anti-rabbit IgG, AF555 donkey anti-rat IgG) for 2 h at room temperature in a dark, humidified chamber. To minimize autofluorescence, the sections were treated with TrueBlack® Lipofuscin Autofluorescence Quencher (Biotium) for 30–60 min, rinsed in PBS, and mounted with DAPI-containing medium (Vectashield®). High-resolution fluorescence images were captured with a Hamamatsu NanoZoomer 2.0 RS equipped with a 40 × objective lens (NA 0.75) at 455 nm/pixel resolution, using a Hamamatsu LX2000 Lightning exciter. All images were taken using two fluorescence channels (488 nm and 561 nm laser lines).

## Results

Patients included in this study had previously undergone karyotyping, MLPA, and CMA, none of which revealed disease-causing genomic alterations. To further investigate potential explanations for the phenotype, exome sequencing was performed to identify small variants. In four patients, only variants of uncertain significance (VUS) were detected (Table 1). However, in patient ST-4, a variant in *SLC13A5* (NM_177550:c.425C > T:p.T142M) was identified. ST-4 is a female patient presenting with epilepsy, delayed speech, intellectual disability, and cerebral atrophy. She exhibits specific facial features, including synophrys, underdeveloped ears, dental issues, gingival hypertrophy, and microdontia. This variant has been reported as pathogenic in the literature (developmental and epileptic encephalopathy 25 with amelogenesis imperfecta, OMIM 615905) and shows a strong overlap with the described clinical features (Hardies et al. 2015).

**Table 1:** Exome sequencing results

Sample	Gene	Variant	ACMG	Disease
CRZ-1	CASK	NM_001367721:c.334G > T,p.V112L	VUS	Intellectual developmental disorder and microcephaly with pontine and cerebellar hypoplasia (OMIM 300749)
CRZ-2	HUWE1	NM_031407:c.3973-2A > G	VUS	Intellectual developmental disorder, X-linked syndromic, Turner type (OMIM 309590)
ST-2	SCN8A	NM_001330260:c.1475G > A:p.R492H	VUS	Cognitive impairment with or without cerebellar ataxia (OMIM 614306), Developmental and epileptic encephalopathy 13 (OMIM 614558)
	ASH1L	NM_018489:c.4927C > T:p.R1643W	VUS	Intellectual developmental disorder, autosomal dominant 52 (OMIM 617796)
ST-3	ASLX3	NM_030632:c.3310G > T:p.E1104*	VUS	Bainbridge-Ropers syndrome (OMIM 615485)
	ATP1A1	NM_000701:c.1573G > T:p.G525C	VUS	Charcot-Marie-Tooth disease, axonal, type 2DD (OMIM 618036), Hypomagnesemia, seizures, and impaired intellectual development 2 (OMIM 618314)
ST-4	SLC13A5	NM_177550:c.425C > T:p.T142M	Pathogenic	Developmental and epileptic encephalopathy 25, with amelogenesis imperfecta (OMIM 615905)

From long-read DNA sequencing, a total of two hundred eighteen genomic regions were analyzed, and 340 SVs were detected. Based on standard genetic screening methods, including karyotyping, MLPA, CMA, and NGS exome sequencing that showed negative results, we could exclude gross (larger than 5 Mb) SVs from further analysis (Supplemental Table S3). Insertions were analyzed separately; two could be excluded, and five met criteria for further analysis (Supplemental Table S4). After filtering, 35 prioritized SVs were subjected to expanded analysis (Supplemental Table S5). In total, 26 candidate SVs were selected, of which 4 appear in more than one patient (Table 2).

**Table 2:**
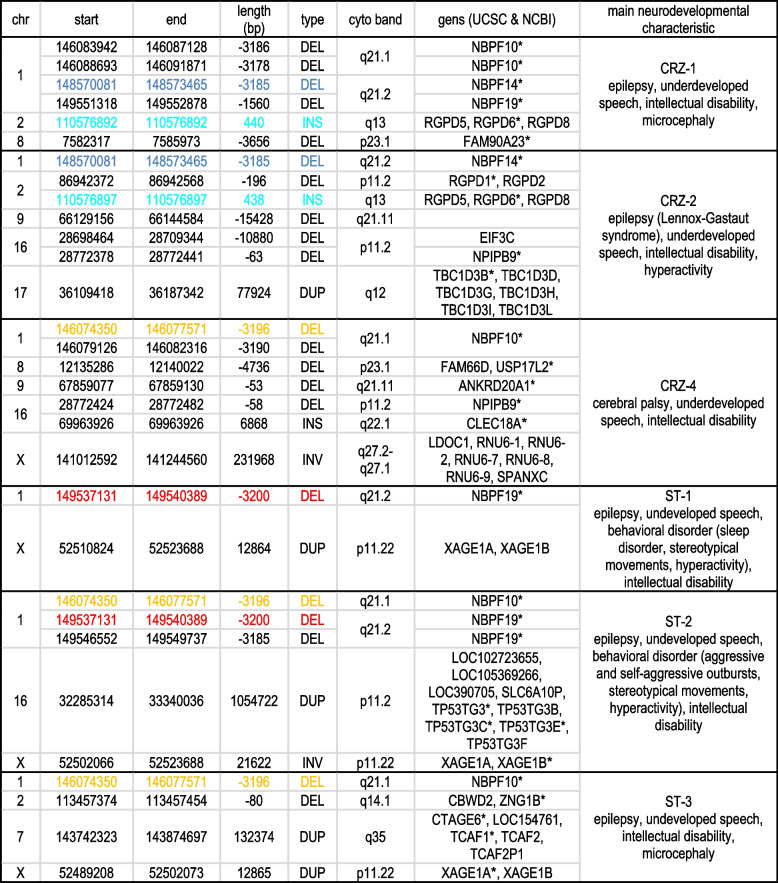
Candidate SVs. A total of twenty-six candidate SVs have been filtered. Five SVs appear in more than one patient and are marked with matching colors. Yellow shows a deletion at NBPF10, detected in patients CRZ-4, ST-2, and ST-3. Dark blue represents a deletion at NBPF14, identified in both CRZ-1 and CRZ-2. Red marks a deletion at NBPF19, observed in ST-1 and ST-2. * denotes MANE transcript

### FISH analysis results

**Fig. 2:**
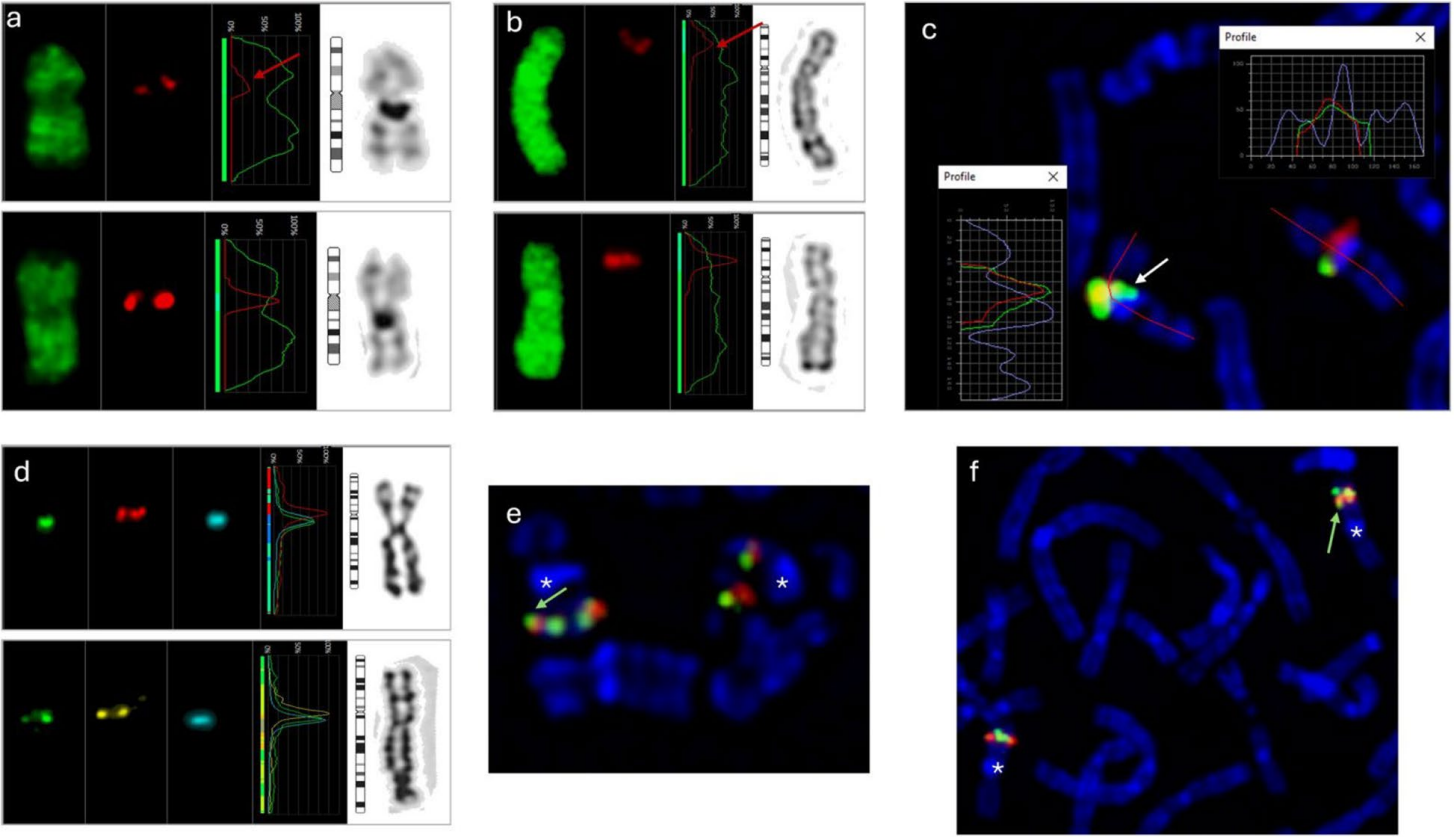
FISH analysis results. FISH results. **a** Patient CRZ-2 deletion on chromosome 16, FISH probe CTD-2555L18 (chr16:28,657,123–28,881,301, UCSC) in red and WCP 16 in green. The upper side picture shows a chromosome that harbors deletion and red arrow indicates 50% signal reduction on chromosome that harbors deletion. **b** Patient CRZ-4 deletion on chromosome 8, FISH probe RP11-351I21 (chr8:12,085,516–12332294, Ensembl) in red and WCP 8 in green. The upper side picture shows a chromosome that harbors deletion and red arrow indicates 50% signal reduction on chromosome that harbors deletion. **c** Patient ST-2, duplication on chromosome 16. FISH probes RP11-17M15 (chr16:32,271,501–32377669, UCSC) in red and RP11-378C4 (chr16:32,979,232–33,067,159, UCSC) in green. White array showing duplication. **d** Patient CRZ-4 according to sequencing results inversion on chromosome X from 52,433,310 to 55,652,522. On the left side, FISH probes RP11-655E2 (chrX:53,393,935–53,558,779, UCSC) in red, RP11-292J24 (chrX:54,777,712–54,949,717, UCSC) in green, and centromere X in blue. On the right side, FISH probes RP11-258C19 (chrX:53,113,469–53,291,902, UCSC) in yellow, RP11-292J24 (chrX:54,777,712–54,949,717, UCSC) in green, and centromere X in blue. Both show normal results. **e** Case ST-4 and **f** case CRZ-4 insertion from the 16q23.1 at 16q22.1 position. In (**e**) and (**f**) FISH probes RP11-142E6 (chr16:70,071,591–70222997, UCSC) 16q22.1 in red and RP11-252A24 (chr16:74,300,891–74464246, UCSC) 16q23.1 in green. In booth cases white array indicates insertion segment from 16q23.1 above 16q22.1. Centrome is marked with the asterix *

Based on available FISH probes (Supplemental Table S6) and the size of the SVs, four SVs could be confirmed, and one could be excluded from future analysis. In two patients, CRZ-2 and CRZ-4 SV deletions were confirmed (Fig. 2a and 2b), whereas in patient ST-2, tandem duplication was confirmed (Fig. 2c). In case CRZ-4, according to sequencing results, there is an inversion in chromosome X from 52,433,310 to 55,652,522. The workflow, which included FISH analysis, enabled us to determine that this inversion was absent in this case (Fig. 2d). Structurally, insertions are interesting SVs, and FISH can provide a factual biological background. In the ST-4 (Fig. 2e) and CRZ-4 (Fig. 2f) cases, an insertion that was detected in 16q22.1 was from the distal part of 16q23.1. In addition, according to the BLAT results, the inserted segment has parts of regulatory elements, including the *GH16J074406* promoter and TTS *LOC105376772* (Supplemental Tables S4 and S7). The GH16J074406 promoter has several related genes, including two protein-coding genes, *CLEC18B* and *NPIPB15*. The functional element LOC127884519 is a gene target for the *GH16J074406* promoter. This element was validated as an active enhancer by the ChIP-STARR-seq massively parallel reporter assay in naive and primed human embryonic stem cells. H3K27ac and H3K4me1 histone modifications characterize this enhancer (Barakat et al. 2018). According to the GeneCards database, the GH16J074406 promoter targets several long noncoding RNAs (lncRNAs). In other cases, the insertions were too small (ranging from 110 to 440 bp) to be detected by FISH probes.

### Candidate SV and interactome analysis results

CRZ-1 is a male patient who exhibits neurodevelopmental features, including epilepsy, completely underdeveloped speech, intellectual disability, and microcephaly. Additionally, he presents with incontinence, strabismus, perceptive hypoacusis, and tetraparesis dystonia as the main clinical findings at the time of examination. Six candidate SVs are detected, and four are located in the NBPF gene family (Table 2, Supplemental Table 7, Supplemental Figure S1a). Deletion in 1q21.1 includes the MANE transcript of the *NBPF10* gene and covers exons 58–61 and 64–66. Next, deletion in 1q21.2 includes the MANE transcript of the *NBPF14* (exons 20–24) and *NBPF19* (exons 90–92) genes. Insertion at 2q13 affects the MANE transcript of the *RGPD6* and the site of the *GH02J110575* promoter and a CpG island (Table 2, Supplemental Table 7, Supplemental Figure S1a). BLAT analysis of the 440 bp inserted segment revealed that the inserted segment maps to the *GH02J087824* promoter, suggesting ectopic promoter activity or regulatory element displacement (Supplemental Table 4). The deletion at 8p23.1 disrupts exon 1 of the *FAM90A23* gene, which belongs to the *FAM90A* multigene family. While deletions in this region have been reported in the DGV Gold Standard at frequencies up to 4.40%, they are absent from gnomAD SV datasets, suggesting their rarity (Supplemental Figure S1a, Supplemental Table 7, 9, 10). For CRZ-1, interactome analysis showed that in the HIPPIE-based interactome (Supplemental Figure S1b), *NBPF10* and *NBPF14* converge on *RGPD6* via the intermediary NEK4, a NIMA-related kinase. The protein encoded by this gene is a serine/threonine protein kinase that is necessary for normal entry into replicative senescence (Nguyen et al. 2012). The encoded protein also plays a role in cell cycle arrest in response to double-stranded DNA damage (Pavan et al. 2021). Interestingly, STRING network analysis did not detect any inter-SV gene connectivity (Supplemental Figure S1c), suggesting that interaction network sensitivity can vary across datasets and interaction inference methods.

**Fig. 3:**
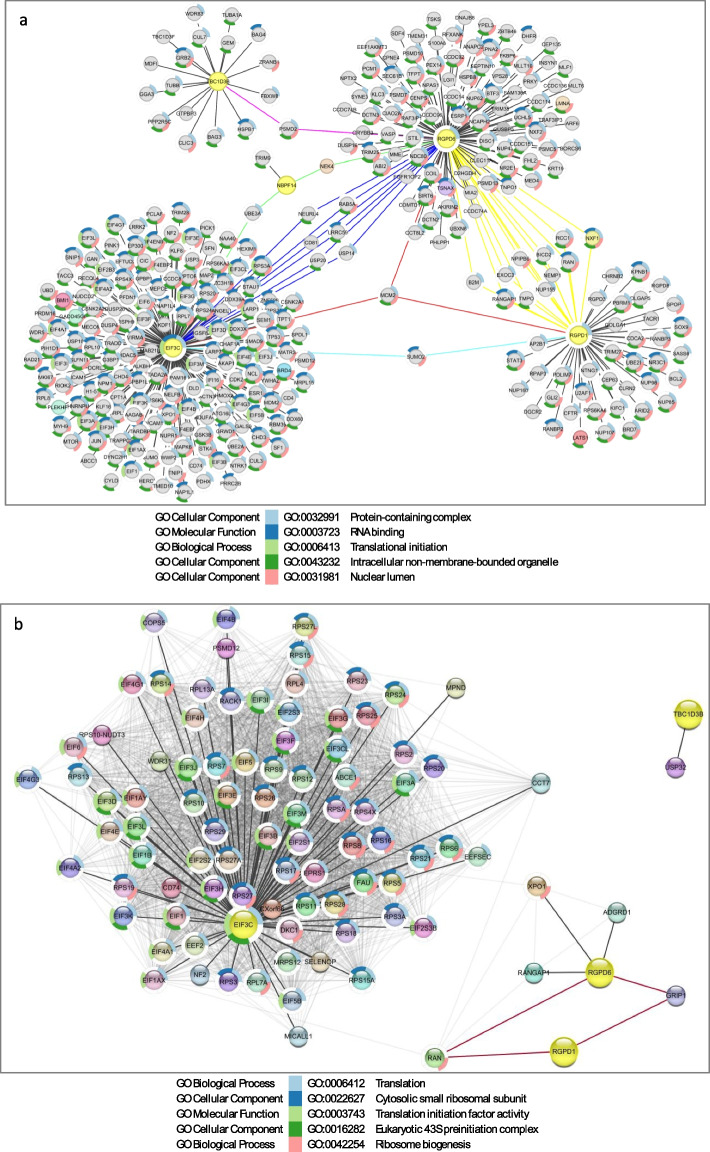
CRZ-2 patient-specific interactome. **a**, Interactome based on HIPPIE database interactions, displays multiple interconnected subnetworks centered around *RGPD6*, *RGPD5*, and *EIF3C*. The RGPD6 network highlights numerous direct interactors, with colored annotations indicating GO terms, including protein-containing complex (blue), RNA binding (purple), translational initiation (green), intracellular non-membrane-bounded organelle (aqua), and nuclear lumen (red). Blue edges emphasize shared interactors between *RGPD6* and *EIF3C*, while yellow and red edges show interactions shared with *RGPD5* and other RGPD6-specific partners. Notably, *RGPD6* bridges networks associated with RNA metabolism and intracellular transport. *EIF3C*, another central hub, is densely connected to ribosomal proteins and translational regulators, indicating its role in translation initiation. **b** interactome derived from the STRING database, depicts a high-confidence interaction network focused on *EIF3C* and ribosomal proteins, emphasizing translation-related processes. *RGPD6* appears in a separate subnetwork, interacting with nuclear transport-related proteins such as *RANGAP1*, *XPO1*, and *RAN*, as well as *RGPD1* and *GRIP1*. GO term annotations include translation (blue), cytosolic small ribosomal subunit (aqua), translation initiation factor activity (green), eukaryotic 43S preinitiation complex (purple), and ribosome biogenesis (red). These networks suggest a potential role for RGPD family members in linking nucleocytoplasmic transport with translational regulation. The redundancy cutoff was set to 0.5. Genes from candidate SVs are marked yellow in (**a**) and (**b**)

CRZ-2 is a female patient who exhibits epilepsy (Lennox-Gastaut syndrome), underdeveloped speech, intellectual disability, and hyperactivity. Additionally, facial asymmetry is present. In total, seven SVs are filtered. Two of them are shared with the CRZ-1 case (Table 2), including deletion at 1q21.2, with the MANE transcript of the *NBPF14* (exons 20–24) and insertion at 2q13 affecting the MANE transcript of the *RGPD6* and the site of the *GH02J110575* promoter and a CpG island (Table 2, Supplemental Table 7, Supplemental Figure S2a). BLAT analysis of the 438 bp inserted segment revealed that the inserted segment maps to the *GH02J087824* promoter, suggesting ectopic promoter activity or regulatory element displacement (Supplemental Table 4). Additionally, a deletion in 2p11.2 (chr2:86,942,372–86,942,568, 196 bp), which covers the MANE transcript of the *RGPD1* and the *GH02J086942* enhancer, which targets the *piR-34097–001*, *NDUFB4P5*, *RGPD1*, and *PLGLB1* genes, was detected. No deletions are reported in the DGV gold standard or gnomAD CNV (Supplemental Figure S2a, Supplemental Tables S9 and S10). This deletion covers a CpG island. Next, deletion at 9q21.11 (chr9:66,129,156–66,144,584) encompasses two TSSs: *piR-30937–010* and *RNU6-538P* (Supplemental Table 7). This 9q21.11 deletion is shared with the ST-4 case. Two deletions affecting 16p11.2 are detected. The chr16:28,698,464–28,709,344 deletion encompasses multiple TTSs but lacks a MANE transcript. NM_001199142 (ENST00000566501.5) represents transcript variant 3 of the *EIF3C* gene within the deletion range (Supplemental Figure S2a). According to the DGV gold standard, five deletions are reported, with frequencies ranging from 0.02% to 8.82% and inner ranks of 13 and 7 (Supplemental Table S9). In the gnomAD CNV dataset, one deletion was documented: 194650_DEL (Supplemental Table S10). *EIF3C* encodes a core subunit of the eukaryotic translation initiation factor 3 (eIF3) complex. Additionally, minor deletions on 16p11.2 impact exon 7 of the MANE transcript of the *NPIPB9* gene were detected (Supplemental Figure S2a). According to the DGV gold standard, one deletion is documented with a frequency of 0.02% and an inner rank of 13. Furthermore, the gnomAD CNV database indicates one deletion (Supplemental Tables S9 and S10). Finally, duplication at 17q12 (chr17:36,109,418–36187342, 77.924 kb) was detected. The duplicated region includes the MANE transcript of the *TBC1D3B* gene. Several TTSs are present, including multiple alternative splicing events and other variations (Supplemental Figure S2a). Additionally, four elite enhancers (*GH17J036123*, *GH17J036132*, *GH17J036148*, and *GH17J036154*) and three elite enhancers/promoters (*GH17J036149*, *GH17J036176*, and *GH17J036182*) are present. A full list of gene targets is available in Supplemental Table 21, indicating that the duplicated segment is transcriptionally active and likely involved in complex gene regulation*. TBC1D3B* is part of the TBC1D3 gene family, a group of hominoid-specific genes that expanded in the human lineage. In the DGV gold standard, multiple CN gains are reported, with frequencies ranging from 0.20% to 23.40% and inner ranks 7 to 13 (Supplemental Table S9). In gnomAD CNV, no data are available for the range chr17:36,109,418–36187342 (Supplemental Table S10). CRZ-2 showed a densely connected HIPPIE-based interactome (Fig. 3 and Table 3).

**Table 3:** Summary for densely connected HIPPIE-based interactome for CRZ-2 case

Interaction	Key Gene	Function/Relevance	Expression Pattern (HBT)	Potential Pathways/Impact
*EIF3C, RGPD1, RGPD6* converge on *MCM2*	*MCM2*	Subunit of the MCM2–7 helicase complex; DNA replication licensing, initiation, and elongation; maintains genomic stability; promotes stem cell differentiation and is required for neural differentiation in mouse ESCs (Ebstein et al. 2012; Yang et al. 2021)	Highly dynamic; peak prenatal expression in ventricular/subventricular zones & hippocampal dentate gyrus (8–24 PCW), rapid postnatal decline	DNA replication, cell cycle control, early neurogenesis
NBPF14 links *EIF3C* ↔ *RGPD6* via *UBE3A* & *NEK4*	*UBE3A*	E3 ubiquitin ligase; tags proteins for degradation; implicated in Angelman & Prader–Willi syndromes (Ebstein et al. 2021)	Broad prenatal expression	Ubiquitin–proteasome–mediated protein homeostasis
	*NEK4*	Serine/threonine kinase; implicated in neurogenesis	Moderate prenatal expression	Neurogenesis regulation
*TBC1D3B* ↔ *RGPD6* via *PSMD2*	*PSMD2*	26S proteasome non-ATPase subunit; degrades ubiquitinated proteins; essential for protein homeostasis (Henneberg and Schulman 2021; Küry et al. 2017; Cuinat et al. 2024); linked to NDDs in other proteasome subunits (Lee 2025; Li et al. 2024)	Moderate–high prenatal expression in cortical & subcortical proliferative/differentiating zones (8–24 PCW), decreases after birth	Proteasome-mediated protein turnover during neurogenesis
*RGPD1* ↔ *EIF3C* via *SUMO2*	*SUMO2*	Posttranslational modifier; SUMOylates transcription factors (SOX1–3, SOX6, Bmi1, Nanog, Foxp1, Mecp2, MEF2A, SOX10) regulating NSC proliferation/differentiation (Bernstock et al. 2019; Bertke et al. 2019; Rodriguez et al. 2019; Cai et al. 2018; Lee et al. 2010)	High expression in early–mid fetal brain; decreases postna^16,17^ tally but remains elevated	SUMOylation control of neurogenesis
*RGPD6* ↔ *EIF3C* via *USP14, USP20, CD81, LRRC59, RAB5A, NEURL4*	*USP14, USP20*	Deubiquitinating enzymes; remove ubiquitin from substrates to stabilize proteins; dysregulation implicated in cancer, neurodegenerative diseases, autophagy, immunity, viral infection (Wang et al. 2021; Qin et al. 2022; Chen et al. 2024, 2007; Hadjebi et al. 2008)	Moderate–high prenatal expression; sustained into adulthood	Ubiquitin–deubiquitin regulation of protein stability
	*CD81*	Tetraspanin; cell adhesion, immune signaling, exosome biology	Stable expression	Membrane trafficking, signaling
	*LRRC59*	Leucine-rich repeat protein; nuclear import and ribosome biogenesis	Stable/moderate expression	Nuclear trafficking
	*RAB5A*	GTPase; early endosome trafficking	Prenatal > postnatal expression	Vesicular trafficking in neurodevelopment
	*NEURL4*	E3 ubiquitin ligase; centriole biogenesis, mitochondrial regulation	Moderate prenatal expression	Organelle biogenesis
*RGPD6* ↔ *RGPD1* via *NPIPB6, RCC1, NXF1, BICD2, B2M, RANGAP1, NUP155, RAN, EXOC3, NEMP1, TMPO*	*NPIPB6*	Nuclear pore interacting protein; limited functional data	Low–moderate expression	Possible nuclear transport
	*RCC1*	Guanine nucleotide exchange factor for Ran; regulates mitosis, spindle organization (Hao and Macara 2008; Punta et al. 2012; Mitchell et al. 2010; Preston et al. 2019)	High prenatal expression, sharp postnatal drop	Cell cycle, chromosome segregation
	*NXF1*	mRNA export factor	Stable/moderate expression	mRNA transport
	*BICD2*	Dynein adaptor; axonal transport	Stable expression	Intracellular trafficking
	*B2M*	β2-microglobulin; immune component, synaptic pruning	Postnatal increase then plateau	Immune–neural interface
	*RANGAP1*	Ran GTPase-activating protein; nucleocytoplasmic transport	Stable expression	Nuclear transport
	*NUP155*	Nucleoporin; nuclear pore structure; pluripotency regulation; knockout affects miR290-295 cluster(Preston et al. 2019)	High prenatal expression, postnatal decline	Nuclear transport, stem cell regulation
	*RAN*	Small GTPase; nucleocytoplasmic trafficking	Stable expression	Transport of proteins/RNA
	*EXOC3*	Exocyst complex subunit; vesicle targeting/exocytosis	Stable expression	Synaptic vesicle release
	*NEMP1*	Nuclear envelope membrane protein; cell division	Stable expression	Nuclear structure
	*TMPO*	Thymopoietin isoforms; progenitor proliferation, chromatin organization (Fleischer et al. 2003; Harris et al. 1995)	High prenatal expression, sharp postnatal drop	Cell cycle, nuclear architecture

CRZ-4 is a male patient diagnosed with cerebral palsy, severely underdeveloped speech, intellectual disability, spastic diparesis (primarily right), and feet in equines. Two deletions in 1q21.1 affecting the MANE transcript of *NBPF10* were detected. Next, the deletion at 8p23.1 (*chr8:12,135,286–12,140,022*) affects exon 1 of the *USP17L2* gene. While deletions in this locus are present in the DGV Gold Standard at variable frequencies (0.21–25.76%), they are absent in the gnomAD Copy Number Variant database, indicating rarity (Supplemental Table 9, 10). Next, as in the CRZ-2 case, minor deletions in 16p11.2 were detected, affecting exon 7 of the MANE transcript of the NPIPB9 gene (Supplemental Figure S3b). According to the DGV gold standard, one deletion is documented with a frequency of 0.02% and an inner rank of 13. Furthermore, the gnomAD CNV database indicates one deletion (Supplemental Tables S9 and S10). Insertion detected at chromosome 16 is located in exon 12 of the MANE transcript of the *CLEC18A* gene. BLAT results of the inserted segment map to the genomic locations of the elite promoters *GH16J074406* targeting *CLEC18B and NPIPB15*; and *GH16J070187* targeting *CLEC18C* and *EXOSC6.* Lastly, an inversion at Xq27.2–q27.1 (*chrX:141,012,592–141244560*) encompasses the *LDOC1* and *SPANXC* genes, as well as elite regulatory elements *GH0XJ141219* and *GH0XJ141176* (Supplemental Figure S3b). STRING network analysis did not detect any direct inter-SV gene connectivity (Supplemental Figure S3c).

ST-1 is a male patient diagnosed with epilepsy, absence of speech, behavioral disorder (sleep disorder, stereotypical movements, and hyperactivity in childhood), intellectual disability, and immobility at the time of the diagnosis. Additionally,incontinence, gastroesophageal reflux disease, hiatal hernia, phimosis, and facial characteristics of underdeveloped nasolabial folds, horizontal eyebrows, cryptorchidism, and hypertonic-hypertrophic musculature were diagnosed. Deletion at 1q21.2 spanning exons 72–92 of the MANE transcript of the *NBPF19* gene was detected. Additionally, multiple cassette exons are present in the detected deletions (Supplemental Figure S4a). No deletions are reported in the DGV gold standard or gnomAD CNV (Supplemental Tables S9 and S10). Inversion in Xp11.22 encompasses the MANE transcript of the *XAGE1B* gene, and several alternative splicing events and other modifications are observed in the inverted region. Interactome analysis did not reveal any direct inter-SV gene connectivity (Supplemental Figure S4b and S4c).

ST-2 female patient presented with epilepsy, undeveloped speech, behavioral disorder (including aggressive and self-aggressive outbursts, stereotypical movements, hyperactivity), and intellectual disability. Additional specific movements were characterized by walking with difficulty on a broad base, moving with the torso tilted to the left with short steps, and movements in both the lower and upper limbs that are free but show partial resistance. Three deletions in the *NBPF* genes are detected (Table 2). First in the MANE transcript of the *NBPF10* gene at 1q21.1, including exons 76–80, which is shared with the CRZ-4 and ST-3 case. Next, two deletions in 1q21.2, including a deletion spanning exons 72–92 of the MANE transcript of the *NBPF19* gene that is shared with the ST-1 case, and a second deletion spanning exons 84–87 of the same gene. In both deletions in 1q21.2, multiple cassette exons are present (Supplemental Figure S5a). No deletions are reported in the DGV gold standard or gnomAD CNV (Supplemental Tables S9 and S10). The 16 p11.2 duplication encompasses three MANE transcripts: *TP53TG3*, *TP53TG3C*, and *TP53TG3E* (Supplemental Figure S5a). Furthermore, the duplication range includes numerous TTSs, alternative splicing events, and CpG islands (Supplemental Table 7, Supplemental Figure S5b). Several deletions are reported in the DGV gold standard, with frequencies ranging from 0.03% to 48.45% and inner ranks of 7–16 (Supplemental Table S9, Supplemental Figure S5b). In gnomAD CNV, no data is available for the chr16:32,285,314–33,340,036 range (Supplemental Table S10, Supplemental Figure S5b). Inversion involving a part of the Xp11.22, ranging from 52,502,066 to 52,523,688, includes the MANE transcript of the *XAGE1B* gene, and several alternative events (Supplemental Figure S5c). No data for the general population are present for this region (Supplemental Table 9, 10; Supplemental Figure S5c). Interactome analysis did not reveal any direct inter-SV gene connectivity in the ST-2 case (Supplemental Figure S5d).

ST-3 case is a female patient diagnosed with epilepsy, absent speech, intellectual disability, complete immobility, spastic extremities, short stature, and microcephaly. Four candidate SVs are detected in the ST-3 case. One SV deletion is detected in the MANE transcript of the *NBPF10* gene at 1q21.1, including exons 76–80, which is shared with the CRZ-4 and ST-2 cases (Table 2). Next, a deletion at chr2:113,457,374–113,457,454 (80 bp) spans the ZNG1B gene and the MANE transcript, including the GH02J113456 enhancer and the gene targets *ZNG1B*, *FOXD4L1*, *LINC02936*, *HSALNG0018062*, and *HSALNG0146574*. No deletion at this location is reported in the DGV gold standard or gnomAD CNV (Supplemental Figure S6a, Supplemental Tables S9 and S10). Duplication on chromosome 7 encompasses two MANE genes, *CTAGE6* and *TCAF1* (Supplemental Figure S6a), including a CpG island and two promoters: *GH07J143796* and *GH07J143835*. In addition, 21 TTSs are present in the region. The gene targets for the elite *GH07J143835* promoter/enhancer are *LOC112267988*, *LOC154761*, *piR-40227–002*, *HSALNG0061974*, *lnc-OR2F2-1*, *TCAF1*, and *CTAGE6*. Multiple alternative splicing events and other variations are also present within the duplicated region (Supplemental Figure S6a). Seven duplications are reported in the DGV gold standard, with frequencies ranging from 0.02% to 4.80% and inner ranks from 7 to 14. In gnomAD CNV, three duplications are reported (Supplemental Figure S6a, Supplemental Tables S9 and S10). This could indicate that 7q35 at the site of the duplication remains a rapidly evolving region with variable copy number in the general population. The contribution of this regulatory-rich region to the phenotype in this particular case is still challenging, as further functional studies need to be done. Duplication in Xp11.22 was detected that includes the MANE transcript of the *XAGE1B* gene. Additionally, several alternative splicing and other events are included, and no SV duplications are reported in this region in the general population (Supplemental Figure S6a, Supplemental Table S7, S9, and S10). Interactome analysis did not reveal any direct inter-SV gene connectivity in the ST-2 case (Supplemental Figure S6b).

### Expression pattern of ANKRD20A1 in the developing cortex of the human frontal lobe throughout the midfetal period

**Fig. 4:**
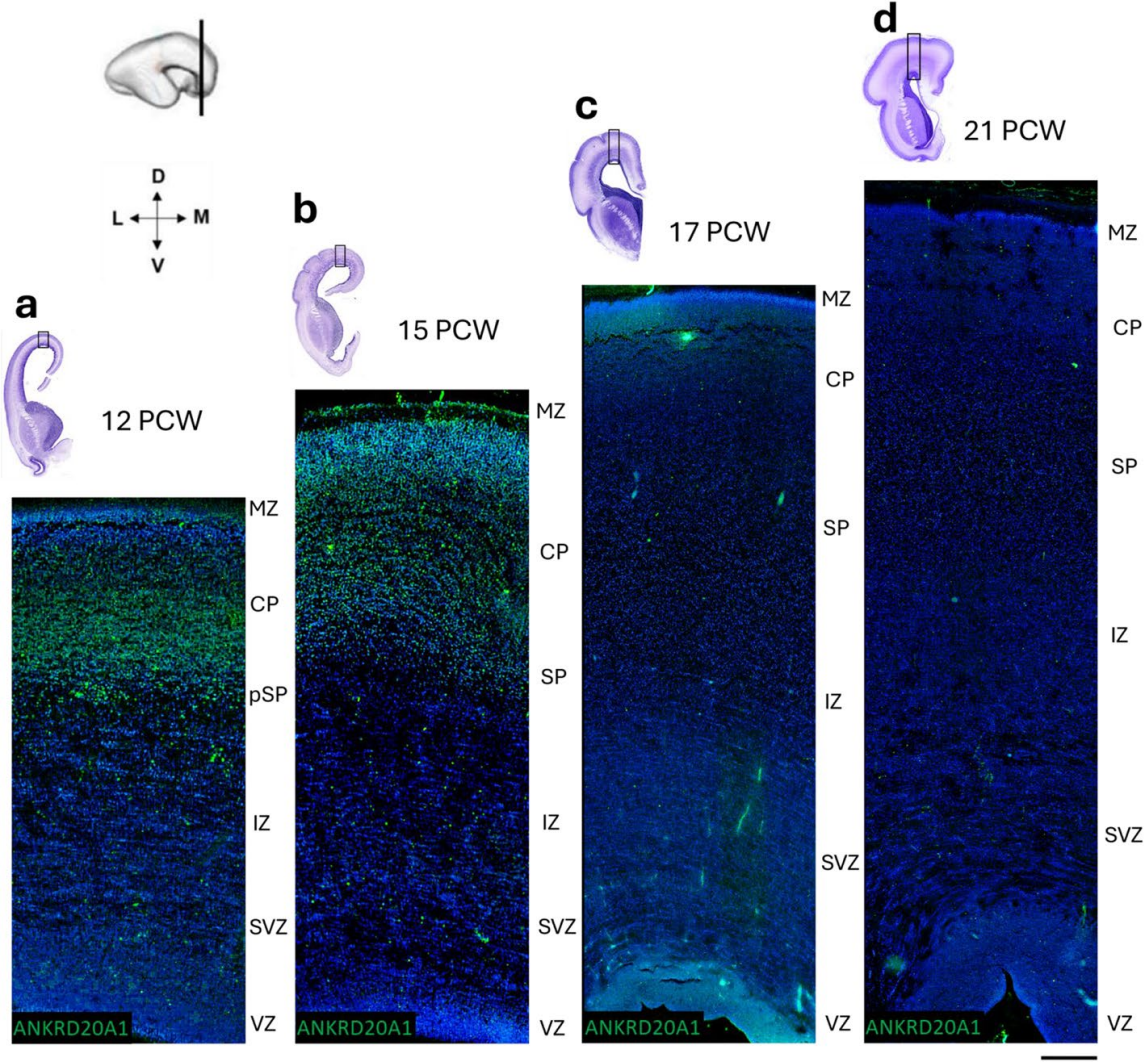
Expression pattern of *ANKRD20A1* in the developing cortex of the human frontal lobe. Dynamic laminar changes in the expression pattern of *ANKRD20A1* (green) in the developing cortex of the human frontal lobe throughout midfetal period (12 PCW—21 PCW). **a**-**d**. Nuclear stain DAPI is shown in blue. Nissl-stained coronal sections (above) show the dorsal portion of the frontal cortex that has been analyzed using immunofluorescence staining. MZ- marginal zone, CP – cortical plate, pSP-presubplate, SP – subplate, IZ – intermediate zone, SVZ – subventricular zone, VZ – ventricular zone.. Scale bar = 200 μm

**Fig. 5:**
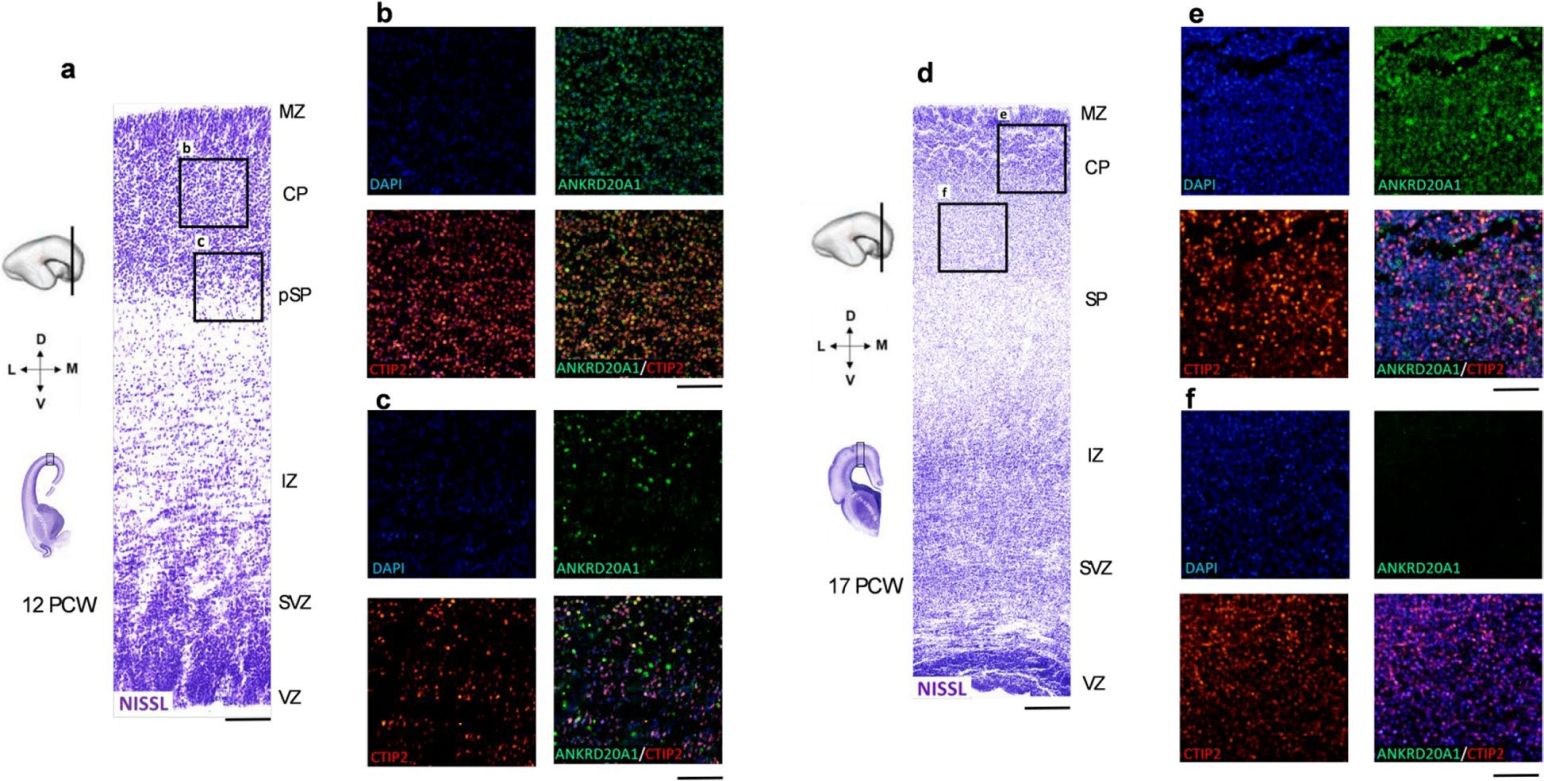
Colocalization of *ANKRD20A1* and *CTIP2* staining in the developing cortex of the human frontal lobe during midgestation. The coronal section through the developing human fetal cortex of the frontal lobe shows localized coexpression of *ANKRD20A1* (green) and *CTIP2* (red) markers at 12 PCW (**b**, **c**) and 17 PCW (**e**, **f**). Nissl-stained sections of the dorsal frontal cortex (**a**, and **d**) show parts of CP (**b**, and **e**), pSP (**c**), and SP (**f**) that were magnified and analyzed with *ANKRD20A1* and *CTIP2* immunofluorescence staining. Nuclear stain DAPI is shown in blue. MZ- marginal zone, CP – cortical plate, pSP-presubplate, SP – subplate, IZ – intermediate zone, SVZ – subventricular zone, VZ – ventricular zone. Scale bar **a**, d = 200 μm, **b**, **c**, **e**, **f **= 100 μm

Given the gnomAD pLI of *ANKRD20A1* is 0.94, strongly suggesting haploinsufficiency may have pathogenic effects, and SV was detected in patient CRZ-4 diagnosed with cerebral palsy, underdeveloped speech, and intellectual disability, further investigation of this gene was warranted. Currently, little is known about the function of *ANKRD20A1* in the brain during neurodevelopment. Therefore, ANKRD20A1 expression was analyzed in the human brain across different developmental stages. *ANKRD20A1* shows a predominant immunofluorescence signal in the cortical plate (CP) at 12 and 15 postconceptual weeks (PCW) (Figs. 4a and b). Moreover, *ANKRD20A1* immunoreactivity was positive in presubplate (pSP) at 12 PCW and in subplate (SP) at 15 PCW (Figs. 4a and b). During the late mid-fetal stages (17 and 21 PCW), we observed a reduced ANKRD20A1 signal in the CP at 17 PCW (Fig. 4c), and an absence of detectable staining in the developing frontal cortex at 21 PCW (Fig. 4d). The immunofluorescence results showed that ANKRD20A1 colocalized with CTIP2, a marker of deep-layer projection neurons, in most neurons within the CP at 12 PCW. Additionally, some cells in the CP displayed no colocalization and were positive for only one marker—either ANKRD20A1 or CTIP2 (Fig. 5b). In the pSP, only a small number of cells showed colocalization of ANKRD20A1 and CTIP2 (Fig. 5c). In contrast, at later developmental stages, we did not observe colocalization in either the CP (Fig. 5e) or the SP (Fig. 5f).

## Discussion

### Classical genetic testing is necessary for patients with neurodevelopmental disorders to ensure biologically accurate results of sequencing

In genetic studies of NDDs, understanding the genomic structure of detected aberrations is essential for assessing their clinical significance (4). Additionally, prioritizing aberrations to enable an etiological diagnosis is challenging. This, together, represents a pitfall in genetic testing involving sequencing technology that must be addressed to achieve biologically accurate results. We combined karyotyping analysis and FISH with LRS to address this problem. In our study, we included patients with unremarkable genetic findings prior to LRS and used FISH to confirm the LRS results. Gross chromosomal rearrangements (< 5 Mb) detected with LRS could be excluded, as conventional karyotyping analysis revealed normal results in all patients. Next, we employed FISH with BAC probes to analyze the sequencing results. This provides a biological and structural context for interpreting sequencing results. We confirmed in two patients, CRZ-2 and CRZ-4, that SV deletions were present (Fig. 2a and b), whereas in patient ST-2, tandem duplication was confirmed (Fig. 2c). Next, we could exclude one inversion on chromosome X in the CRZ-4 case (Fig. 2d).

Insertions detected by LRS are interesting to study because they could represent a “two-way “ hit model for affecting gene function. Hence, in an initial step, disruption of the coding, sequencing, or regulatory regions was determined. The second “hit” would be that the inserted segment has a regulator or coding sequence that could also affect gene expression. The origin of the inserted segment was identified using BLAT analysis (Supplemental Table 4). We applied this approach to cases CRZ-4 and ST-4, where insertions of ~ 6.8 kb in 16q22.1 were detected (Table 2). Although a pathogenic aberration was identified in the CRZ-4 case, we included it in further FISH analysis to emphasize the importance of using complementary methods to understand structural abnormalities detected by sequencing. In CRZ-4, the insertion (chr16:69,963,926–69,963,926) affects exon 12 of the *CLEC18A* gene, while in ST-4, the insertion site (chr16:69,964,152–69,964,152) is outside of the *CLEC18A* gene. In both cases, the inserted segment maps to 16q22.1 and also to 16q23.1, which includes another c-type lectin gene, *CLEC18B* (Supplemental Fig. 7). To confirm the insertion's origin, FISH was performed with probes specific to the 16q22.1 and 16q23.1 regions. FISH analysis confirmed that the inserted segment originates from the more distal part of 16q23.1 and hence *CLEC18B* (Fig. 2e and 2f; Supplemental Fig. 7). Together, these findings emphasize the importance of karyotyping, FISH, and sequencing in deciphering the genomic structures of aberrations. None of these methods alone can currently determine SV structure in sufficient detail to produce biologically accurate results.

### Candidate SVs for future functional studies

Our analysis identified twenty-six SVs, with four appearing in multiple cases (Table 2). Due to the high homology and repetitive nature of these regions, limited data are available in the literature. Here, we focus on the candidate genes with the strongest literature support to prioritize them for further functional studies. First, gene families detected in multiple cases are discussed, followed by SVs detected in individual cases.

#### *NBPF* gene family

**Fig. 6:**
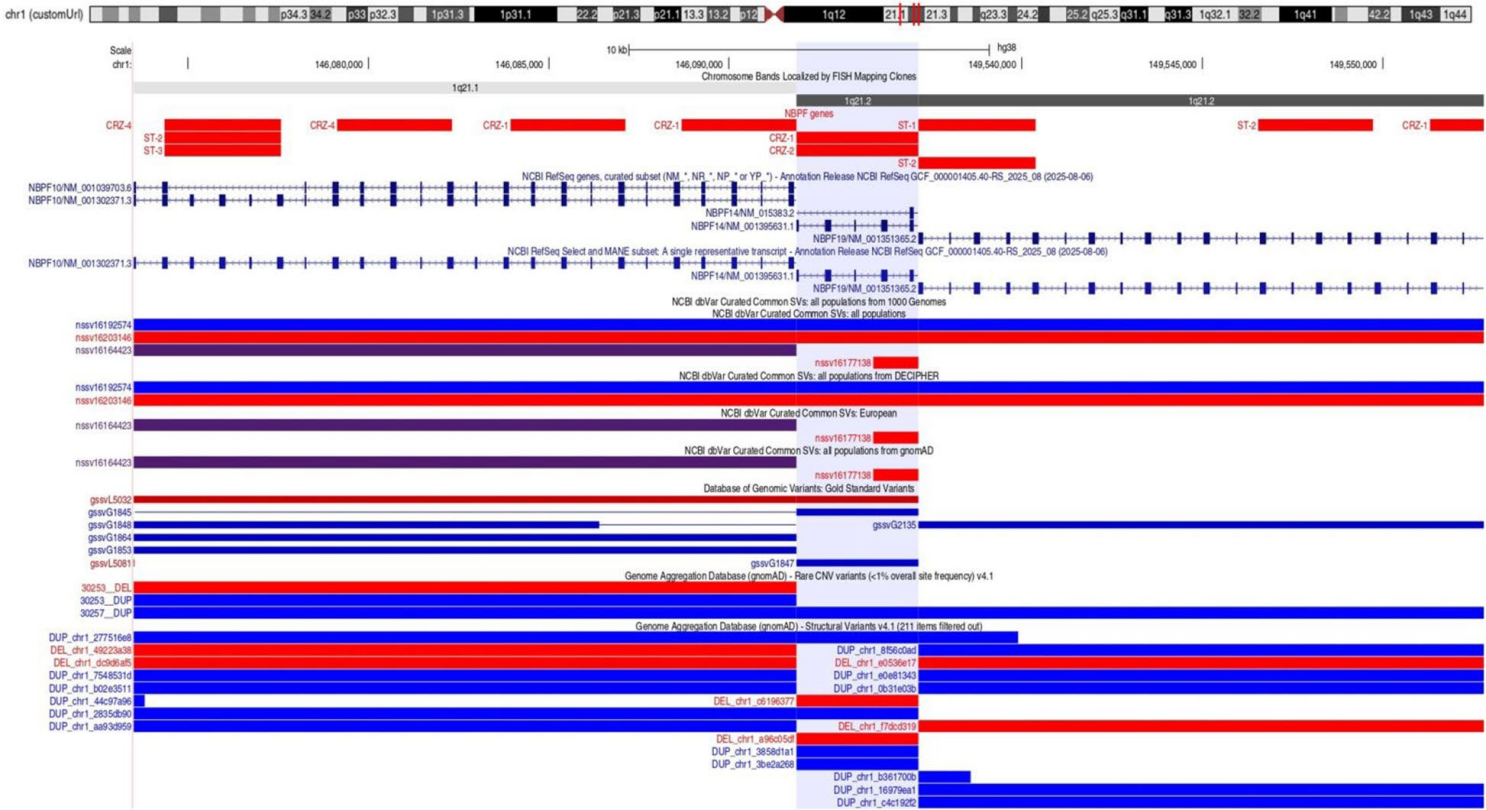
UCSC Genome Browser views of three different NBPF genes (*NBPF10*, *NBPF14*, *NBPF19*) showing gene structure, SV variants detected in the studied patient, and structural variations from dbVar, gnomAD, and DGV gold standard. In red are presented deletions, in blue duplications, and in purple copy number variations (present as deletions and duplications)

Most of the candidates are on chromosome 1 across three *NBPF* gene family members, including *NBPF10*, *NBPF14*, and *NBPF19*. All genes in the NBPF family are built from repeat units. Extensive research has revealed correlations between the copy number of the NBPF genes regions and various factors, including brain size, the number of cortical neurons, IQ scores, cognitive abilities, evolutionary development, and brain disorders such as autism, schizophrenia, microcephaly, macrocephaly, and neuroblastoma (Vandepoele et al. 2005, 2008; Popesco et al. 2006; Dumas et al. 2012; Davis et al. 2014; Quick et al. 2016; Heft et al. 2020). The NBPF gene region has undergone human-specific hyperamplification, which represents the largest human-specific increase in the copy number of any coding region in the genome, with copy numbers generally decreasing with increasing phylogenetic distance from humans: humans have ~ 300 copies, great apes 97–138, monkeys 48–75, and nonprimate mammals 1–8 (Heft et al. 2020). The ~ 1.6-kb NBPF gene regions form canonical 3mer higher-order repeats (HORs) that are highly structured and human-specific (Paar et al. 2011; Glunčić et al. 2023). These tandems occur in four human NBPF genes (*NBPF20*, *NBPF19*, *NBPF10*, *NBPF14*) and show no copy-number variation or mutations across 20 human genomes, although point mutations exist in non-canonical monomers. The T2T assembly identified 61 3mer HORs in humans, but none in chimpanzees or orangutans, and only 9 in gorillas (Glunčić et al. 2024). Overall, tandemly organized 3mer HORs are abundant in humans (36 vs. 0 in great apes), with human NBPF monomers showing twice the divergence observed in apes. Although *NBPF* genes are still under-characterized functionally, their high degree of exon modularity and their location within the highly duplicated 1q21.1 region make them plausible contributors to genomic instability and phenotypic variation. Most recently, *NBPF14* and *NOTCH2NLB*, two coevolved human-specific genes, were shown to coordinately regulate apical progenitor behavior to expand basal progenitors and support neocortical growth central to human cognitive evolution (Eşiyok et al. 2025). Thus, NBPF genes may represent novel candidates for NDD-associated genes and could be involved in the pathogenesis of the patients. Deletions detected in the *NBPF14* (exons 20–24 in CRZ-1 and CRZ-2) and *NBPF19* (exons 90–92 in CRZ-1) genes involve cassette exons, which could impact transcript diversity. Such variations may disrupt mRNA splicing or protein coding potential, affecting the protein makeup of cells (Hakim et al. 2017; Zhang et al. 2019). Data from the general population, available from different sources, indicate that these genomic regions harbor several population-present SVs (Fig. 6). Among the dbVar-curated European SVs, the nssv16164423 SV is reported as a copy-number variant with an allele frequency > 0.5. This SV spans all three NBPF genes. From the DGV gold standard, gssvL5032 deletion is reported with an overall frequency of variants across all studies of 0.0649825%, this deletions span *NBPF10* and *NBPF14*. SVs specific for NBPF10 are reported from the gnomAD—Rare CNV variants (< 1% overall site frequency) v4.1 database, including 30253__DEL, and from the gnomAD—Structural Variants v4.1 database, 49223A38 deletion with allele frequency 0.00015, and dc9d6af5 with allele frequency 0.00016. In *NBPF14* from gnomAD, the rare CNV variant (< 1% overall site frequency), c6196377 deletion, has a frequency of 2.4e-05, and a96c05df has a frequency of 6.4e-05. Only one deletion, f7dcd319, in *NBPF19* is reported in gnomAD—Rare CNV variants (< 1% overall site frequency), with a frequency of 0.05. All this suggests that deletions in the NBPF10, NBPF14, and NBPF19 genes in this region are rare in the general population.

#### *RGPD* gene family

Next, we identified several candidate SVs on chromosome 2, three of which disrupt *RGPD* genes (Table 2). RGPDs originated from partial duplication and fusion of *RANBP2* and *GCC2*, followed by further duplication and gene conversion in apes (58,59). They are ape-specific, with species-specific copy numbers for *RGPD1–8*: gibbon (1), orangutan (3), gorilla (4), chimpanzee (6), and human (7). The only exception is *RGPD7*. A recent study suggests that RGPD copies arose independently in multiple ape lineages, with only *RGPD2* conserved among African great apes. The human-specific *RGPD6* likely emerged from *RGPD5* about 5.2 kya (Mao et al. 2024). The high nucleotide identity between *RGPD5* and *RGPD6* frequently results in inversions and microdeletions, with deletions associated with Joubert syndrome due to the nephronophthisis (*NPHP1*) gene in their intergenic region (Mao et al. 2024; Parisi et al. 2004). All seven RGPDs are expressed in human cells but lack several RANBP2 domains, including the ZF region, RBDs, and the E3 SUMO ligase domain. As RGPDs fuse *RANBP2* with *GCC2***,** they likely localize to the trans-Golgi and may acquire novel functions (Desgraupes et al. 2023). Single-cell RNA data show distinct expression patterns: *RGPD6* in neurons, *RGPD1* in neuronal, glial, and germ cells, and *RGPD2* additionally in blood and immune cells (Human Protein Atlas). SVs encompassing RGPD6-5 and 8 were detected in a patient diagnosed with microcephaly (CRZ-1) (Table 2). Furthermore, interactome analysis revealed that *NBPF10* and *NBPF14*, both affected by SVs in the CRZ-1 case, converge on *RGPD6* via NEK4 (Supplemental Figure S1b). Next, in the CRZ-2 cases, two SVs were identified that impacted the *RGPD1*, *RGPD2*, *RGPD5, RGPD6,* and *RGPD8* genes. An extensive interactome was developed for CRZ-2, revealing that *RGPD1* and *RGPD6*, together with the candidate gene, form the 16p11.2 *EIF3C*, which exhibited a significant number of interactions (Fig. 3 and Table 3). Three candidate genes, *EIF3C*, *RGPD1*, and *RGPD6*, converge on MCM2—a key helicase subunit of the MCM2-7 complex essential for DNA replication licensing and early neurogenesis, with peak expression in proliferative brain zones during prenatal development. Additional links revealed *NBPF14* bridging *EIF3C* and *RGPD6* via *UBE3A* and *NEK4*, implicating ubiquitin ligase–mediated protein degradation and neurogenesis regulation. *TBC1D3B* is connected to *RGPD6* through *PSMD2,* a 26S proteasome component with high prenatal expression, supporting intense protein turnover during neurodevelopment. Other connections included SUMO2, linking *RGPD1* and *EIF3C*, highlighting SUMOylation’s role in neural stem cell proliferation and differentiation. *RGPD6*–*EIF3C* interactions passed through deubiquitinating enzymes (*USP14*, *USP20*) and other genes with sustained prenatal expression, while *RGPD6*–*RGPD1* links involved nuclear transport and cell cycle regulators (*RCC1*, *NUP155*, *TMPO*) with roles in mitosis, nuclear pore function, and progenitor proliferation. Additionally, extensive information regarding this dense HIPPIE interactome is available in Supplemental Figure S2b and S2C. Overall, the CRZ-2 network suggests disruption of two major processes: ubiquitin/deubiquitination–mediated protein homeostasis and cell cycle/molecular trafficking control, both critical for early brain development. Importantly, none of these variants from chromosome 2 were present in the DGV Gold Standard or gnomAD Copy Number Variants datasets, indicating that they are rare and potentially deleterious. As with the *NBPF* genes, *RGPD* genes cannot be fully assessed using short-read sequencing, leading to limited comprehensive analyses and population-level data. This gene family represents an interesting candidate for NDD genes and may contribute to the neuronal pathogenicity observed in patients carrying SVs affecting these loci.

#### Additional candidate genes that were detected only in an individual case

In the CRZ-1 case, the deletion occurred at exon 1 of the *FAM90A23* gene, which is a member of the *FAM90A* multigene family and is situated within a segmentally duplicated and highly polymorphic region of chromosome 8p23. (Bosch et al. 2007). Although the functional characterization of *FAM90A23* is still limited, *FAM90* genes are believed to be involved in cancer development, progression, and immune system regulation, and may also have roles in gene regulation during early development. (Liu et al. 2024; Zhang and Yang 2023). The presence of these genes in dynamic genomic regions suggests susceptibility to copy number changes, yet alterations affecting coding exons, as observed here, may impact gene expression. The deletion overlaps with exon 1, which is critical for transcription initiation and mRNA stability, increasing the possibility of gene dosage or regulatory disruption.

Deletion at 16p11.2 in the CRZ-2 case involves multiple TTSs and NM_001199142 transcript variant 3 of the *EIF3C* gene. Several deletions have been reported in population databases for this deletion range. However, the wide frequency range and inner_rank values are unreliable for predicting how common this region is as a CNV in the general population. *EIF3C* encodes a core component of the eukaryotic translation initiation factor 3 (eIF3) complex, which is crucial for starting mRNA translation. Disruption of translation regulation has increasingly been associated with neurodevelopmental disorders, especially in rapidly dividing neural progenitor cells during cortical development (Mohamed and Klann 2023; Harnett et al. 2022; Borisova et al. 2024; Paul et al. 2023). Next, on chromosome 17, the SV duplication affecting several genes from the *TBC1D3* family is detected in the CRZ-2 case. Included in the duplicated regions are *TBC1D3B*, *TBC1D3D*, TBC1D3G, *TBC1D3H*, *TBC1D3I*, and *TBC1D3L* genes. Among them, the MANE transcript is present only for the *TBC1D3* gene. This region is rich in regulatory elements, suggesting roles beyond *TBC1D3* itself (Supplemental Tables S7, S11). The hominoid-specific *TBC1D3* gene family expanded in humans and is linked to chromosomal rearrangements on chromosome 17 (66). This gene family has independently duplicated in at least five primate lineages, with duplicated loci enriched at sites of large-scale chromosomal rearrangements on chromosome 17 (Guitart et al. 2024). *TBC1D3* promotes dendritic arborization and the pace of synaptogenesis, and the generation of outer radial glia, possibly affecting human cortical neuron development (Dong et al. 2024; Ju et al. 2016; Zhao et al. 2024). Although multiple duplications in this region are reported in the DGV gold standard, all have high inner ranks 7 to 13 (Supplemental Table S9), making them unreliable for sound conclusions about genetic diversity in the general population. This is supported by the absence of CNVs in the gnomAD CNV database (Supplemental Table S10). Future characterization and functional analysis could provide more detailed insights into whether SVs in *TBC1D3B* contribute to neurodevelopmental disorders; however, it is undeniably a promising candidate for further research.

In the CRZ-4 case, several individual SVs are detected. This patient is the only one diagnosed with cerebral palsy. The deletion at 8p23.1 (*chr8:12,135,286–12,140,022*) affects exon 1 of the *USP17L2* gene. *USP17L2* is a member of the polymorphic USP17 deubiquitinating enzyme family. These enzymes regulate protein stability and turnover by removing ubiquitin moieties from target proteins. Ubiquitin signaling has established roles in neural progenitor maintenance, neuronal differentiation, and synaptic connectivity (52–56). The deletion overlaps the transcription start site and first exon, which may cause transcriptional silencing or abnormal initiation. Next, a deletion on chromosome 9 impacts exon 1 of the *ANKRD20A1* gene, encompassing the GH09J067858 promoter, which is directed towards piR-42891–006, ENSG00000306566, ANKRD20A1, and ZNG1C. The *ANKRD20A1* gene encodes a protein containing ankyrin repeat domain motifs, which is essential for its functions mediating protein‒protein interactions to regulate signaling pathways (Li et al. 2006). Although *ANKRD20A1* has not yet been linked to neurodevelopmental processes, other ankyrin repeat-containing genes, such as *ANK3*, *ANK2*, and *SHANK3*, have been implicated in neurodevelopmental disorders, including autism, intellectual disability, and microcephaly (Okuzono et al. 2024; Jiang et al. 2024; Buijsse et al. 2023). Interestingly, single-cell RNA expression analysis revealed that ANKRD20A1 is expressed exclusively in excitatory neurons, oligodendrocyte precursor cells, inhibitory neurons, and germ cells (The Human Protein Atlas). The absence of reported deletions at this locus in population databases, and the pLI, which indicates haploinsufficiency as a pathomechanism, strengthen the hypothesis that the ANKRD20A1 gene is a good candidate for further functional research. Hence, this LRS study on SVs could potentially identify a new strong candidate gene for neurodevelopmental disorders. However, segregation analysis is needed to confirm this. Lastly, an inversion at Xq27.2–q27.1 (*chrX:141,012,592–141244560*) encompasses the *LDOC1* and *SPANXC* genes, as well as elite regulatory elements GH0XJ141219 and GH0XJ141176 (Supplemental Figure S3a). *LDOC1* is a transcriptional regulator and tumor suppressor that modulates NF-κB signaling, a pathway important for neuroinflammatory responses and early stages of neurodevelopment (82). *SPANXC*, part of the SPANX gene family, is primarily testis-expressed, and its role in the brain remains unclear; however, regulatory changes in this region could have broader chromatin-level effects. For CRZ-4, HIPPIE-based interactome analysis (Supplemental Figure S3c) revealed a single notable interaction: *LDOC1* and *USP17L2* are connected via SUDS3, a transcriptional corepressor within histone deacetylase (HDAC) complexes that mediate chromatin remodeling (118). This link suggests a convergence between ubiquitin-mediated protein regulation (*USP17L2*) and epigenetic control (*LDOC1* via SUDS3).

In the ST-2 case, the 16p11.2 duplication encompasses three MANE transcripts: *TP53TG3*, *TP53TG3C*, and *TP53TG3E*, which include numerous TTSs, alternative splicing events, and CpG islands. These genes are members of the TP53TG3 family and are known to be p53-induced transcripts. The tumor suppressor p53 plays a crucial role in embryonic neuron development and neurite growth (Giovanni et al. 2006). p53 regulates synaptic plasticity, reducing neuronal excitability and the number of excitatory synapses in the primary somatosensory cortex (Kuang et al. 2022). Loss of p53 function leads to structural, functional, and synaptic deficits in cortical neurons (61). The functions of *TP53TG3*, *TP53TG3C*, and *TP53TG3E* are not well defined, but the p53 signaling pathway plays key roles in regulating neural progenitor proliferation, cell cycle arrest, and apoptosis during neurodevelopment. Dysregulation of this pathway through dosage-sensitive duplications may alter the developmental trajectory, especially if it persists in early embryonic stages. In the ST-2 case, a severe behavioral phenotype could potentially be influenced by this duplication.

Duplication on chromosome 7 in the ST-3 case was detected, which encompasses two MANE genes, *CTAGE6* and *TCAF1*. In addition, multiple regulatory elements are preset. *CTAGE6* encodes a member of the *CTAGE* protein family, which is involved in intracellular trafficking and may transport proteins from the endoplasmic reticulum (ER) to the Golgi. Although its role in neurodevelopment is not fully understood, intracellular ET-to-Golgi trafficking has been implicated in cellular stress responses and vesicle trafficking (Kim et al. 2023). Disruption of intracellular transport pathways has been recently linked to neurodevelopmental disorders (Xiong and Sheng 2024). *TCAF1* (TRPM8 channel-associated factor 1) regulates the activity and trafficking of the TRPM8 ion channel, which is involved in calcium signaling. While the most well-known functions of *TCAF1* are related to sensory processing, recent studies suggest that ion channel regulation is essential during cortical development, influencing progenitor proliferation, neuronal excitability, and migration (Bando et al. 2022). Dysregulation of calcium homeostasis has been implicated in various neurodevelopmental phenotypes, including cortical malformations and cognitive impairment (Arjun McKinney et al. 2022).

### Developmental expression pattern of ANKRD20A1: implications for cortical architecture

The developmental expression pattern of *ANKRD20A1* was investigated in the developing human brain, as no data are currently available for humans or animal models during prenatal neurodevelopment. Moreover, no animal model for this gene has been identified, and the available literature is extremely limited. Given that single-cell RNA expression data for *ANKRD20A1*, as reported in the Human Protein Atlas, show its expression is largely restricted to excitatory neurons, oligodendrocyte precursor cells, inhibitory neurons, spermatocytes, and spermatogonia—and that this gene is disrupted in a patient diagnosed with cerebral palsy (CRZ-4)—providing information on its spatiotemporal expression during human brain development offers valuable complementary insight. Analysis of *ANKRD20A1* expression in the developing human cortex reveals a dynamic, stage-specific pattern across postconceptual weeks (PCW) with strong signals observed in the cortical plate (CP) at 12 and 15 PCW, suggesting a role in early cortical development. At 12 and 15 PCW, *ANKRD20A1* is expressed in the transient cortical layers—the presubplate (pSP) and subplate (SP)—indicating active involvement during critical developmental stages, likely contributing to early cortical layer formation and the establishment of subcortical connections. By 17 and 21 PCW, *ANKRD20A1* expression progressively decreases and is no longer detected in the frontal cortex by 21 PCW, suggesting a transient role during early neurogenesis. Its partial colocalization with CTIP2, a marker of deep-layer projection neurons, suggests potential involvement in early deep-layer neuron specification. However, the presence of neurons expressing only one marker highlights cellular heterogeneity. Limited colocalization in the presubplate (pSP) and subplate (SP) at later stages further suggests that *ANKRD20A1* is not exclusively associated with deep projection neurons but exhibits a broader, more varied expression profile. Overall, the data indicate that ANKRD20A1 may play a critical role in early cortical development, particularly in the cortical plate and subplate at 12 and 15 PCW. Its transient expression pattern supports a role in early neurogenesis and cortical layer formation, while its interaction with CTIP2 suggests a possible role in neuronal differentiation and the establishment of cortical architecture, underscoring the need for further investigation.

## Conclusion

This study illustrates the utility of integrating long-read DNA sequencing with traditional cytogenetic techniques to elucidate the structural intricacies of genomic variants associated with NDDs. By combining FISH and LRS, we confirmed structural variations previously undetectable by conventional genetic analyses and accurately delineated their genomic architecture, thereby facilitating biologically precise interpretations. Our analysis showed enrichment of rare and potentially deleterious SVs impacting gene families specific to primates, including *NBPF*, *RGPD*, *TBC1D3*, and *ANKRD20A1*—genes implicated in cortical development, cognitive evolution, and language-associated traits. However, for now, all these findings can only be characterized as variants of uncertain significance under current ClinGen classification guidelines. Developmental expression profiling of *ANKRD20A1* revealed transient, stage-specific activity in the fetal cortical plate and subplate, suggesting a potential role in early neurogenesis and cortical layer formation. This supports the sing-cell RNA transcriptomic data from the literature that *ANKRD20A1* is postnatally expressed almost exclusively in neuronal and glial cells. Collectively, these findings broaden the corpus of candidate genes for functional investigation, emphasize the evolutionary perspective of NDDs, and demonstrate how detailed structural variant characterization can uncover potentially novel genetic mechanisms underlying complex neurological phenotypes.

## Limitations of the study

Despite these promising findings, several limitations must be acknowledged. First, the small sample size limits the generalizability of the results and precludes strong genotype‒phenotype correlations. Additionally, although interactome analysis offers insights into potential gene‒gene interactions, these findings are predictive and require experimental validation. The lack of parental samples also hampers the ability to determine whether variants are de novo or inherited. Furthermore, additional transcriptomic and proteomic data would be helpful in further understanding the biological effects of these variants. There are also methodological limitations. Due to the lower coverage (15–20x), bioinformatic analysis of SVs may be less sensitive and reliable compared to high-coverage samples (> 35x). In addition, population metrics for candidate SVs are absent or only partially available for evolutionarily recent gene families, making the pathogenicity assessment of the respective SVs challenging.

## Supplementary Information

Supplementary Material 1.

Supplementary Material 2.

## Data Availability

All data generated or analyzed during this study are included in this published article and its Supplemental information files.

## Abbreviations

SV: Structural variant
NDD: Neurodevelopmental disorder
NGS: Next-generation sequencing
CMA: Chromosomal microarray
MLPA: Multiplex ligation-dependent probe amplification
GS/ES: Genome sequencing / Exome sequencing
FISH: Fluorescence in situ hybridization
IGV: Integrative Genomics Viewer
BAC: Bacterial artificial chromosome
PBS: Phosphate-buffered saline
PCW: Postconception weeks
TSS: Transcription start site
CpG: Cytosine-phosphate-Guanine (nucleotide sequence context)
MANE: Matched Annotation from NCBI and EMBL-EBI
DGV: Database of Genomic Variants
gnomAD CNV: Genome Aggregation Database Copy Number Variant
BLAT: BLAST-Like Alignment Tool
HIPPIE: Human Integrated Protein–Protein Interaction Reference
STRING: Search Tool for the Retrieval of Interacting Genes/Proteins
ncbiRefSeq: NCBI Reference Sequence Database
DAPI: 4′,6-Diamidino-2-phenylindole
CRL: Crown–rump length
HOR: Higher-order repeat
HLS: Hominoid-specific sequences (Olduvai domains)
