# Supplementary material for: Structural variations in evolutionary novel genomic regions: new insights into neurodevelopmental disorders by long-read DNA Sequencing
Source: Mol Med. 2026 Jan 9;32:17. doi: 10.1186/s10020-025-01415-y (PMC12882164; doi:10.1186/s10020-025-01415-y)

Supplemtal figures

Supplementary Figure S1. Structural variants and interactome analysis in patient CRZ-1. (a) Genomic visualization of SVs, including deletions affecting NBPF10, NBPF14, NBPF19, an insertion at 2q13 involving RGPD6 and regulatory elements, and a deletion at 8p23.1 affecting FAM90A23. (b) HIPPIE interactome demonstrating convergence of NBPF10 and NBPF14 on RGPD6 via NEK4. (c) STRING analysis showing no inter-SV gene connectivity, highlighting dataset-specific differences.


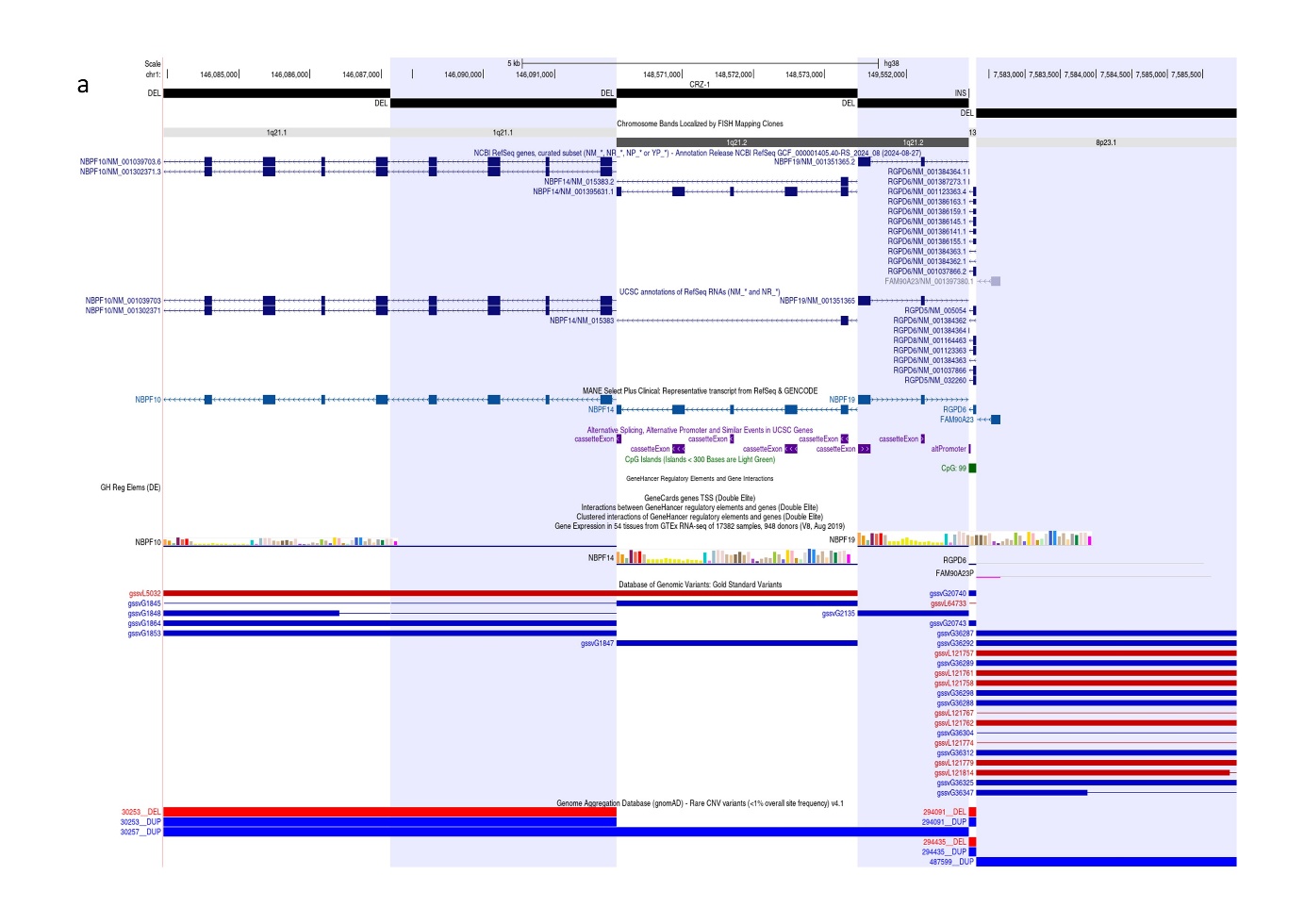

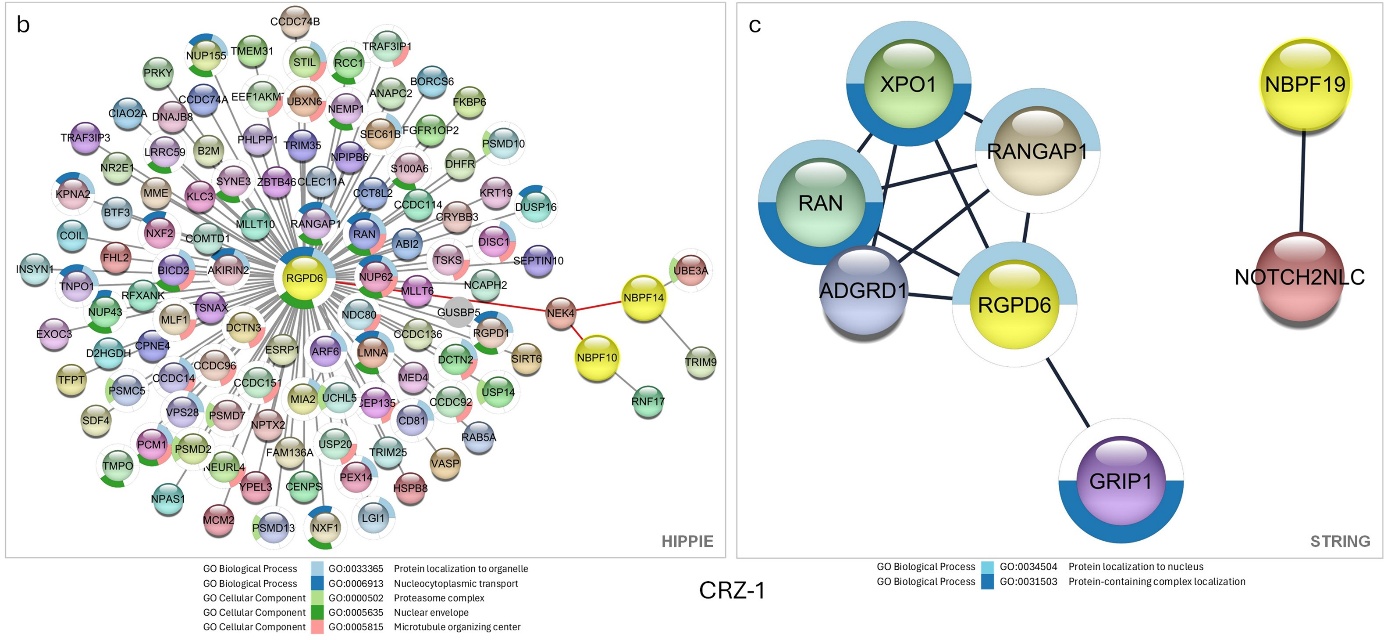


Supplementary Figure S2. Structural variants and networks identified in patient CRZ-2. (a) SVs including NBPF14 deletion, insertions/deletions involving RGPD1, RGPD2, RGPD5, RGPD6, RGPD8, deletions at 16p11.2 (EIF3C and NPIPB9), and a large 17q12 duplication encompassing TBC1D3B family genes. (b–c) HIPPIE-based interactome showing a dense network connecting EIF3C, RGPD1, RGPD6, and NBPF14, whereas STRING reveals a less complex structure.


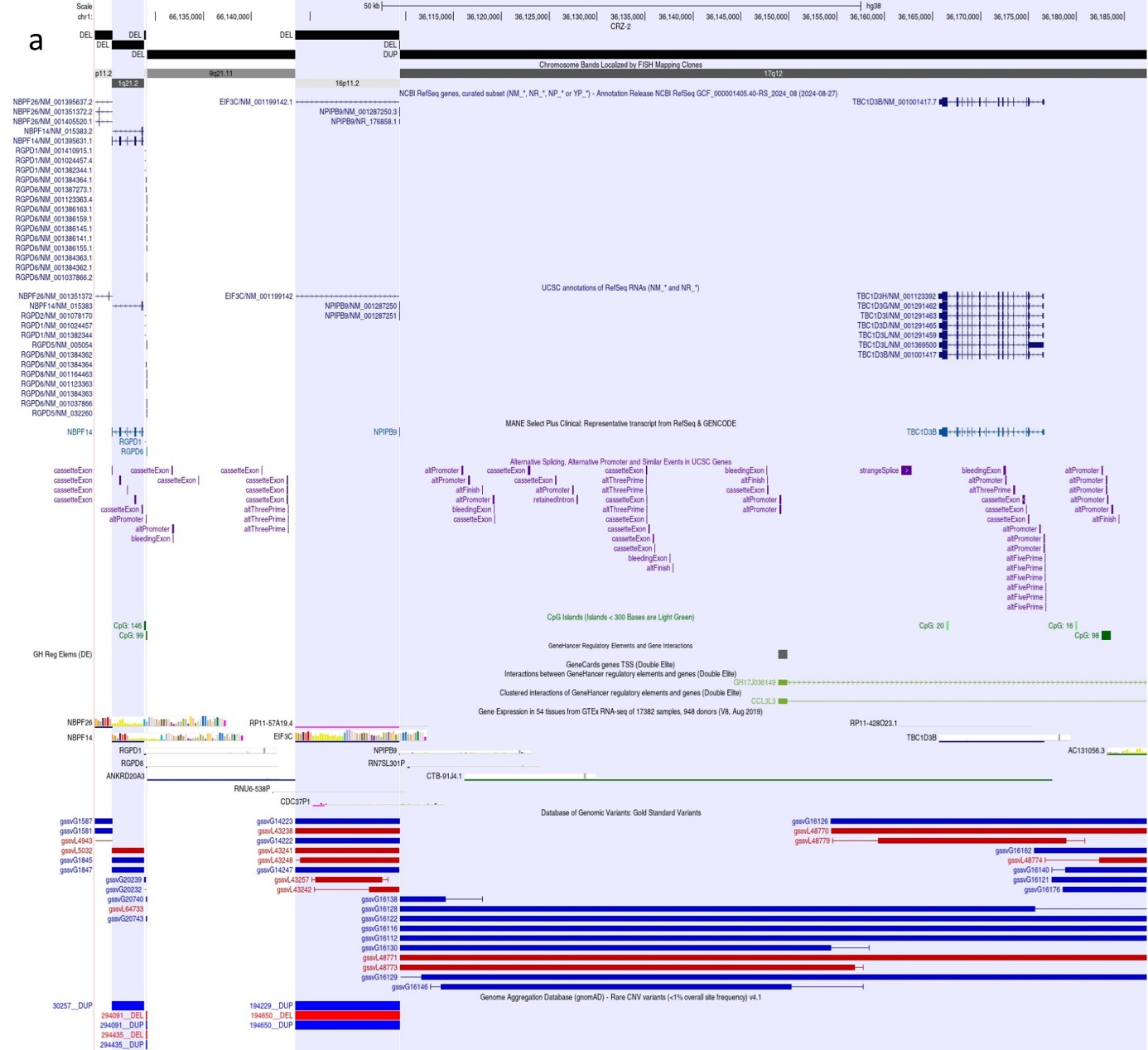


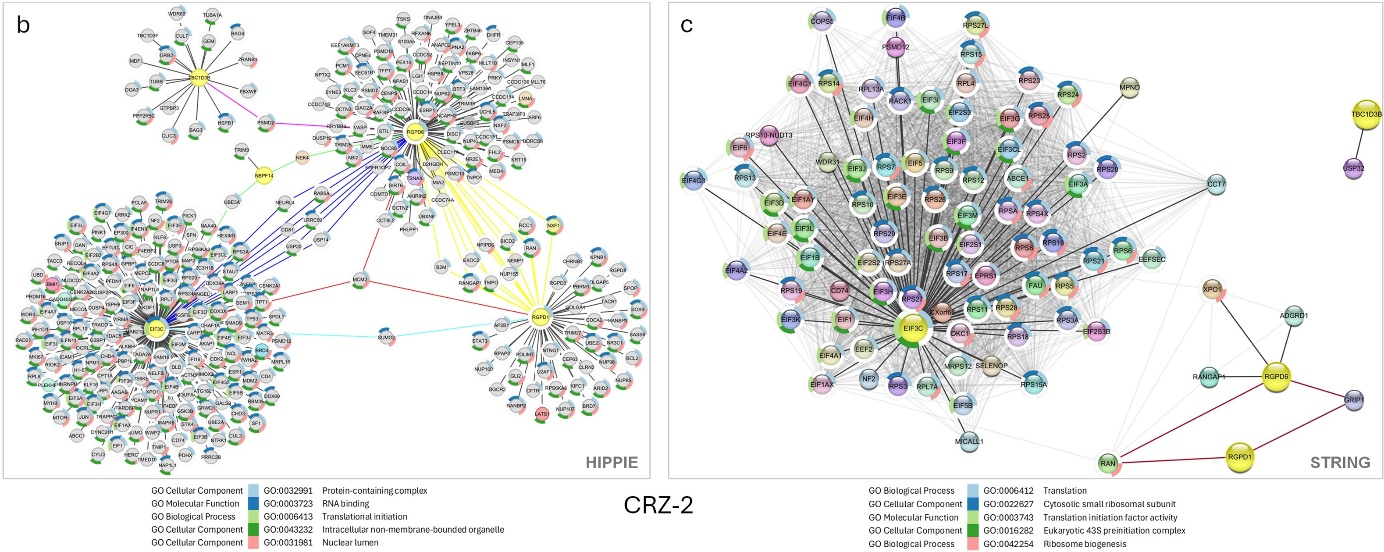


Supplementary Figure S3. Structural variants and interactome overview for patient CRZ-4. (a) SVs including deletions in USP17L2, ANKRD20A1, NPIPB9, a CLEC18A-disrupting insertion, and an Xq27.2–q27.1 inversion encompassing LDOC1 and SPANXC. (b) Genomic visualization of NPIPB9 deletions. (c) HIPPIE interactome showing a singular significant link: LDOC1 ↔ USP17L2 mediated by SUDS3, a key chromatin remodeling corepressor.
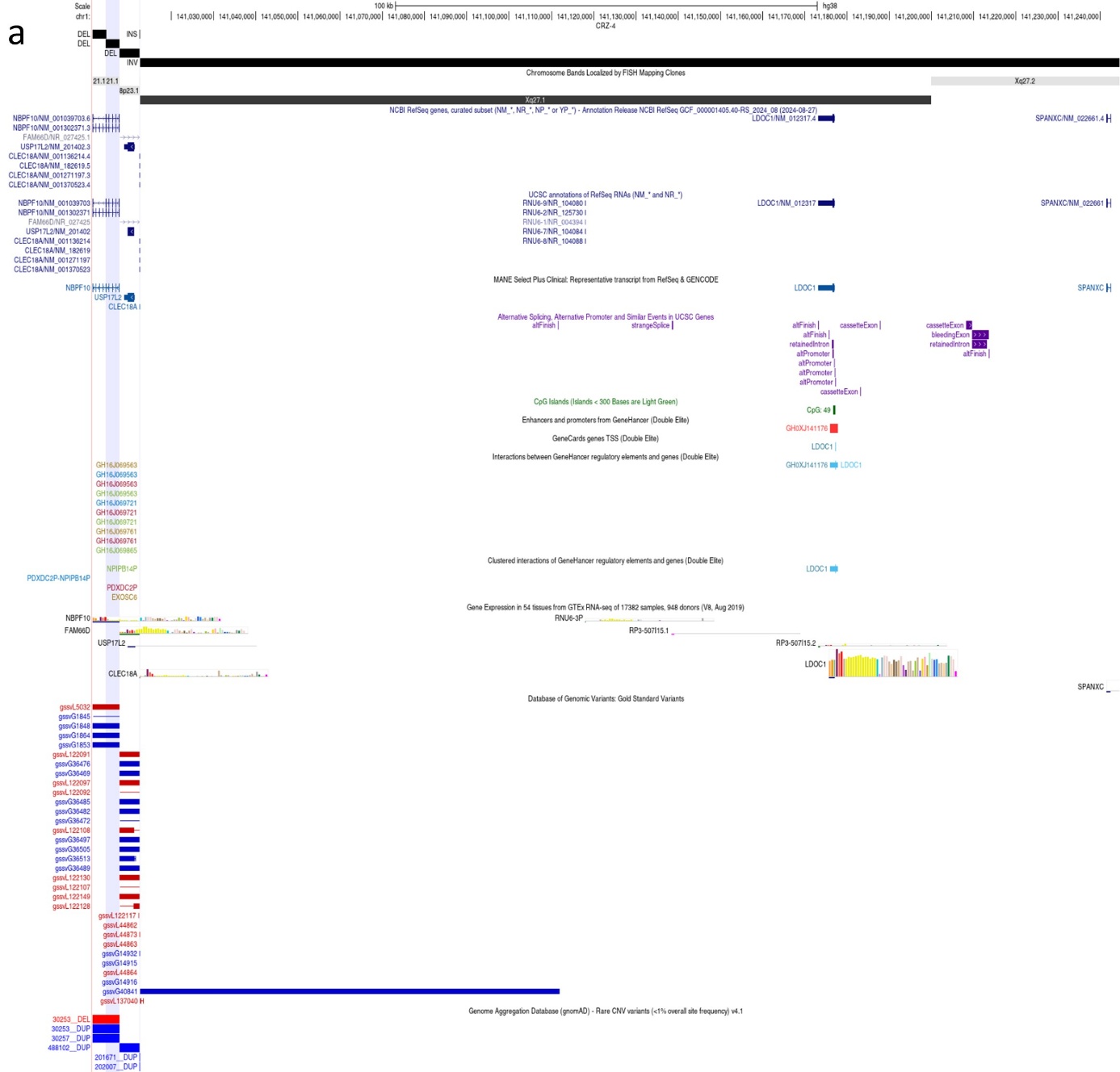


Supplementary Figure S3 continued.


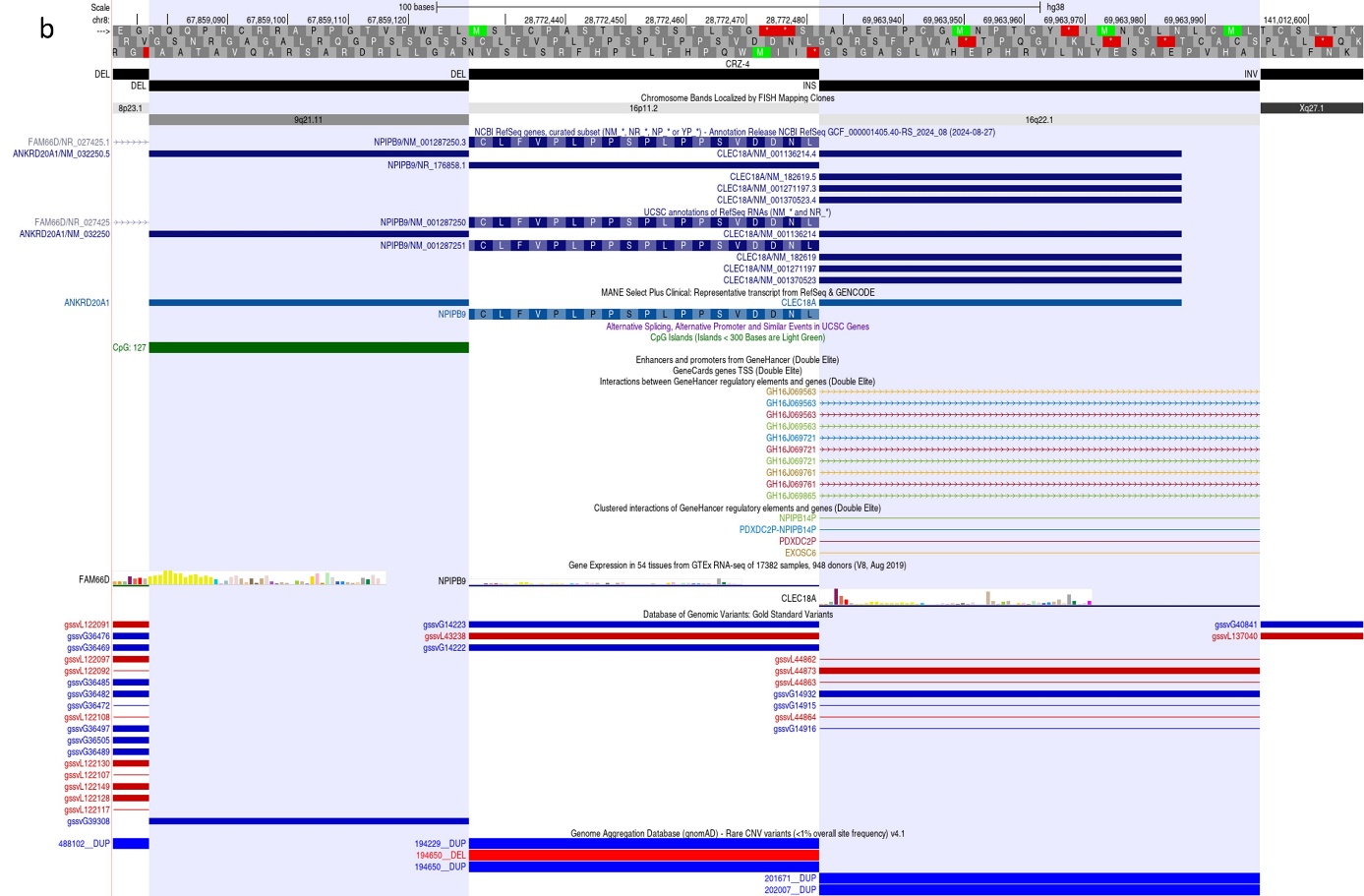


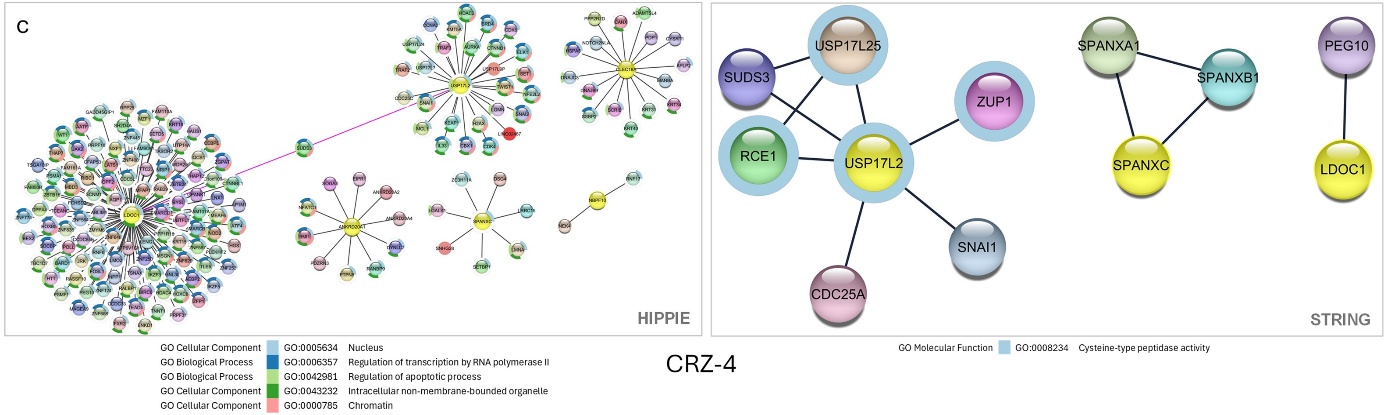


Supplementary Figure S4. Structural variants and interactome results for patient ST-1. (a) SVs include a large NBPF19 deletion and an inversion at Xp11.22 affecting XAGE1B. (b–c) Interactome analyses showing no inter-SV gene connectivity.


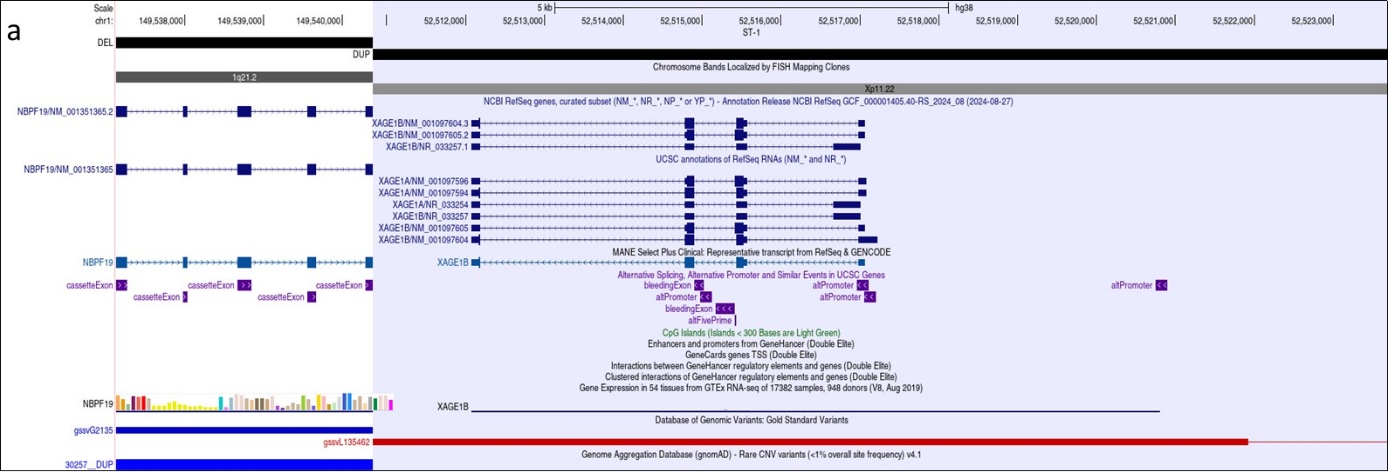

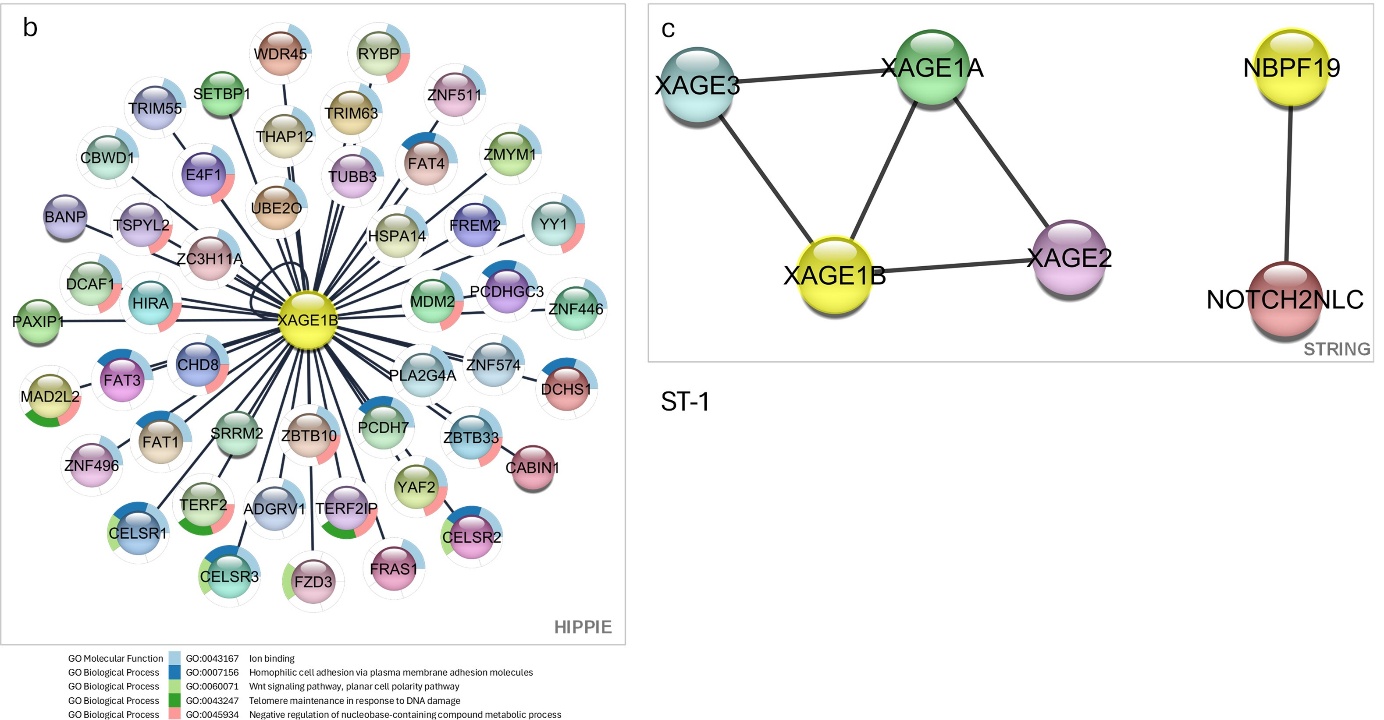


Supplementary Figure S5. Structural variants and interactome results for patient ST-2. (a) NBPF10 and NBPF19 deletions, including shared loci with other cases. (b) 16p11.2 duplication encompassing TP53TG3, TP53TG3C, and TP53TG3E, with extensive regulatory features. (c) XAGE1B inversion. (d) No inter-SV gene interactions detected in interactome analyses.


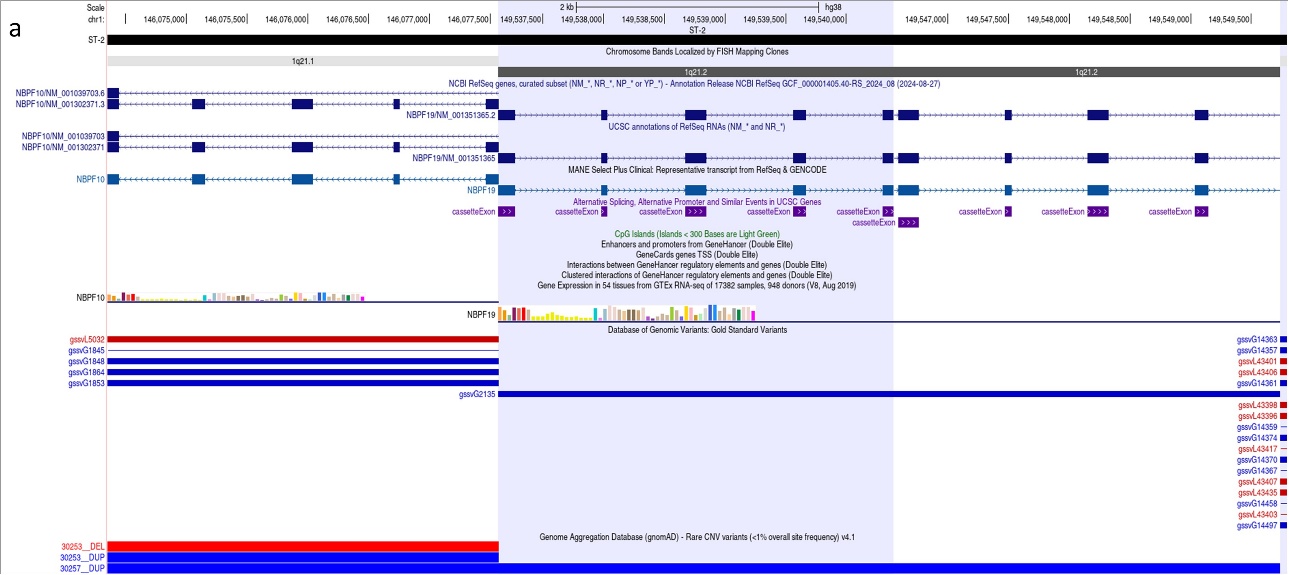

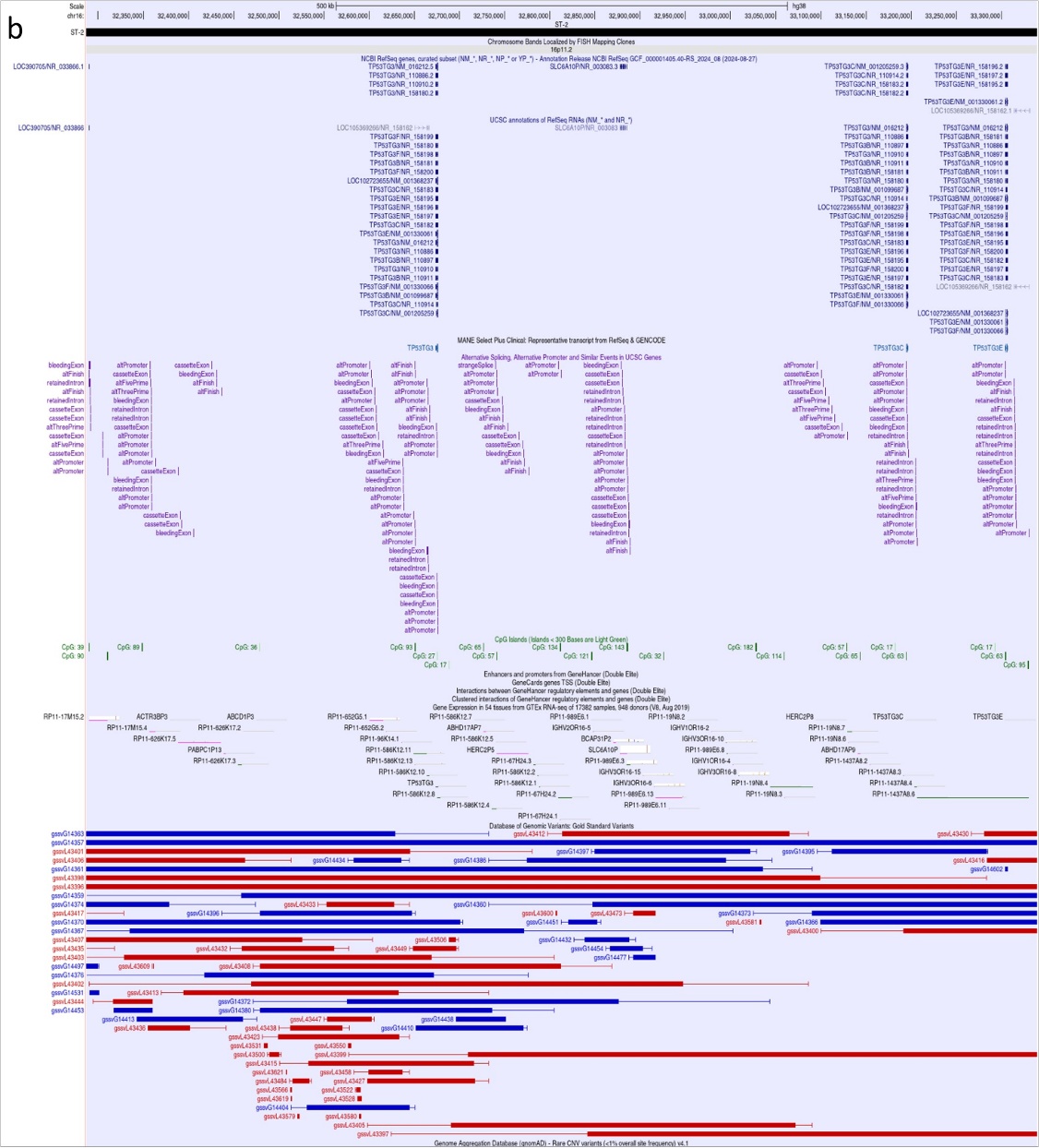


Supplementary Figure S5 continued.


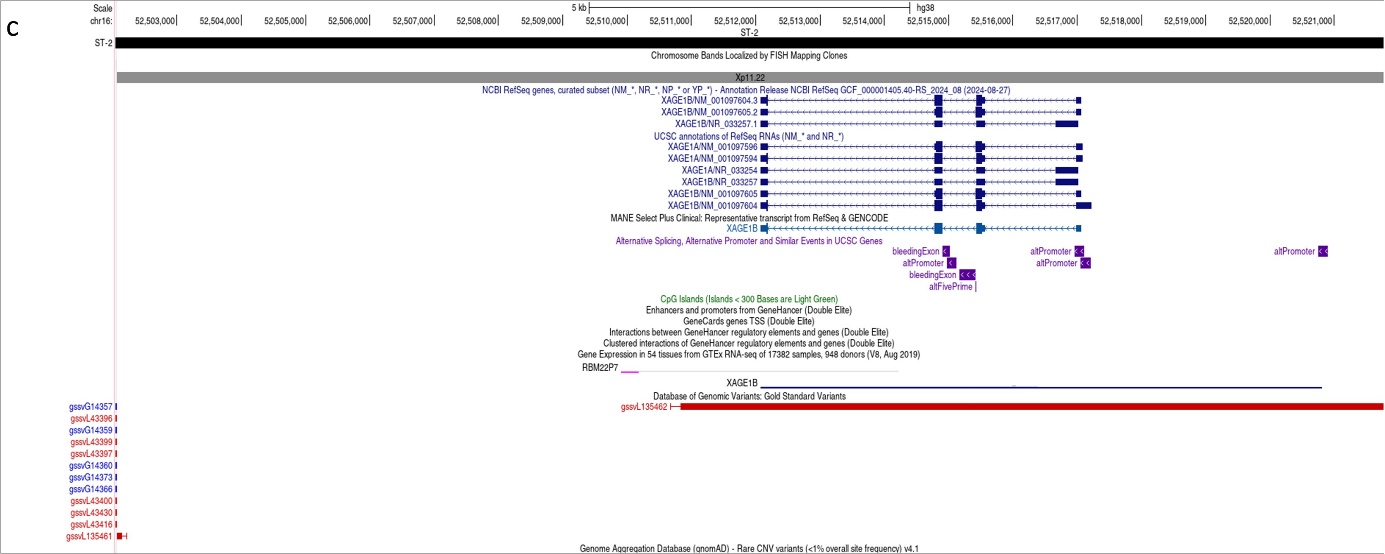


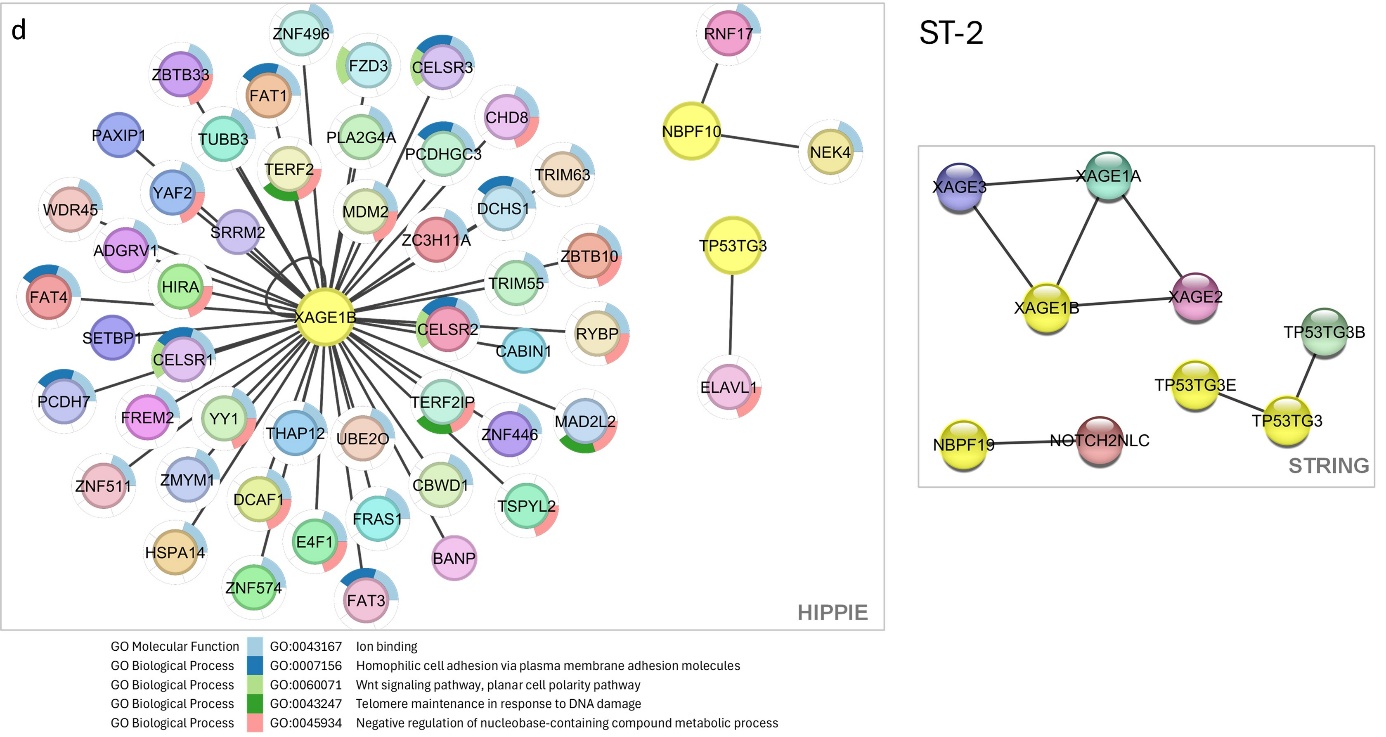


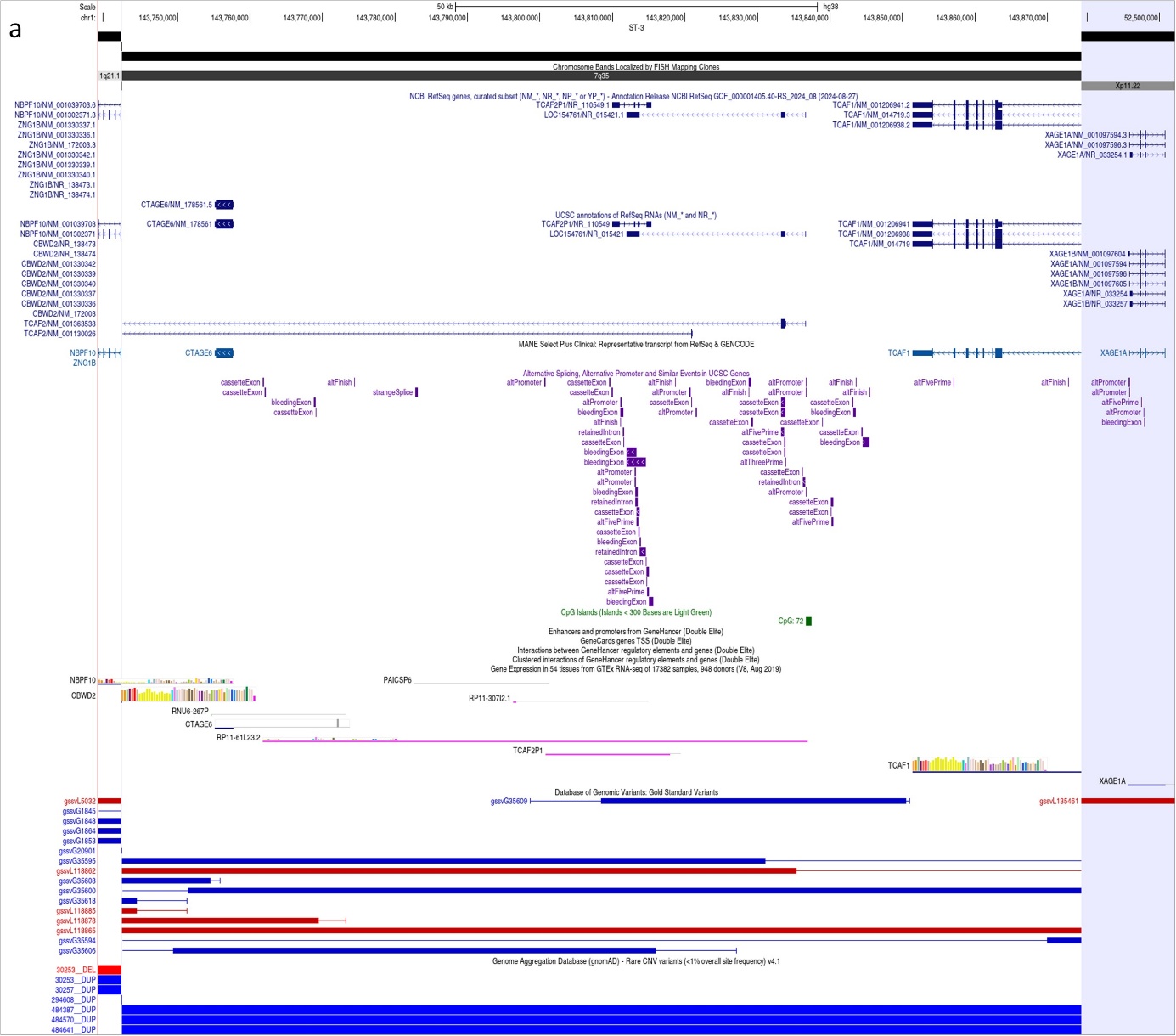
Supplementary Figure S6. Structural variants and interactome results for patient ST-3. (a) SVs include NBPF10 deletion, chr2 enhancer deletion, a chr7 duplication involving CTAGE6 and TCAF1, and duplication of XAGE1B. (b) Interactome analysis showing absence of functional connectivity among SV-affected genes.


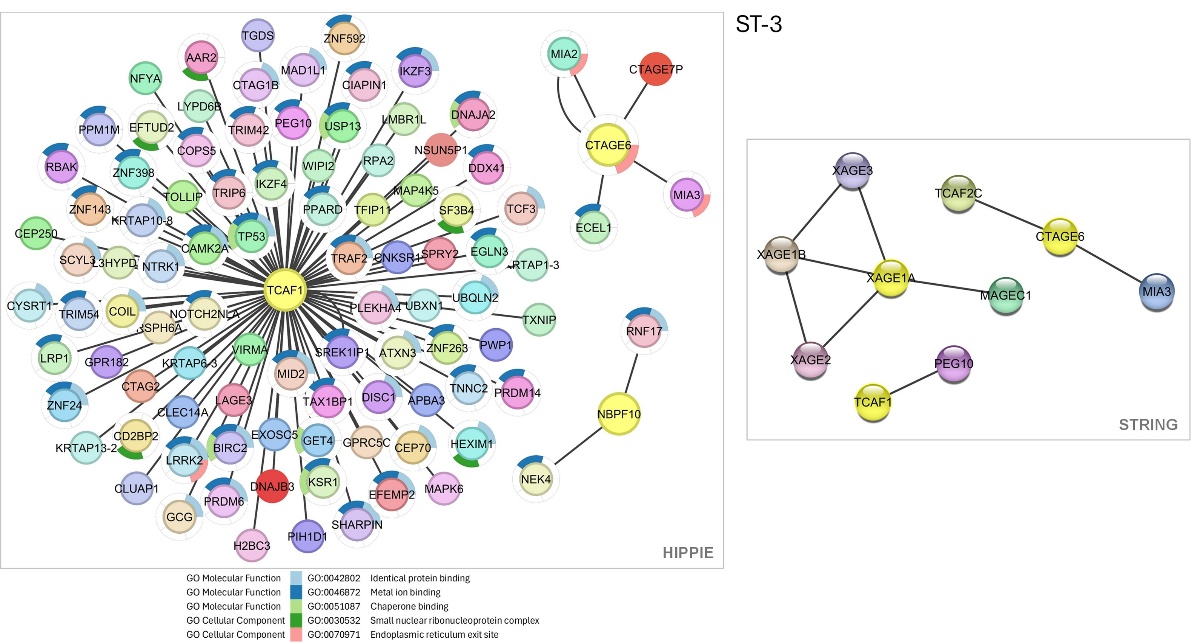


Supplementary Figure S7. Origin of 16q22.1 insertions in patients ST-4 and CRZ-4. (a–c) Genomic alignments and FISH mapping demonstrate that the ∼6.8 kb insertion at 16q22.1 originates from 16q23.1 and contains regulatory elements and C-type lectin family genes (CLEC18B, NPIPB15). FISH probes spanning both loci confirm the distal origin of the insertion. For FISH figures refer to the Figures 2E & 2F in the main manuscript.


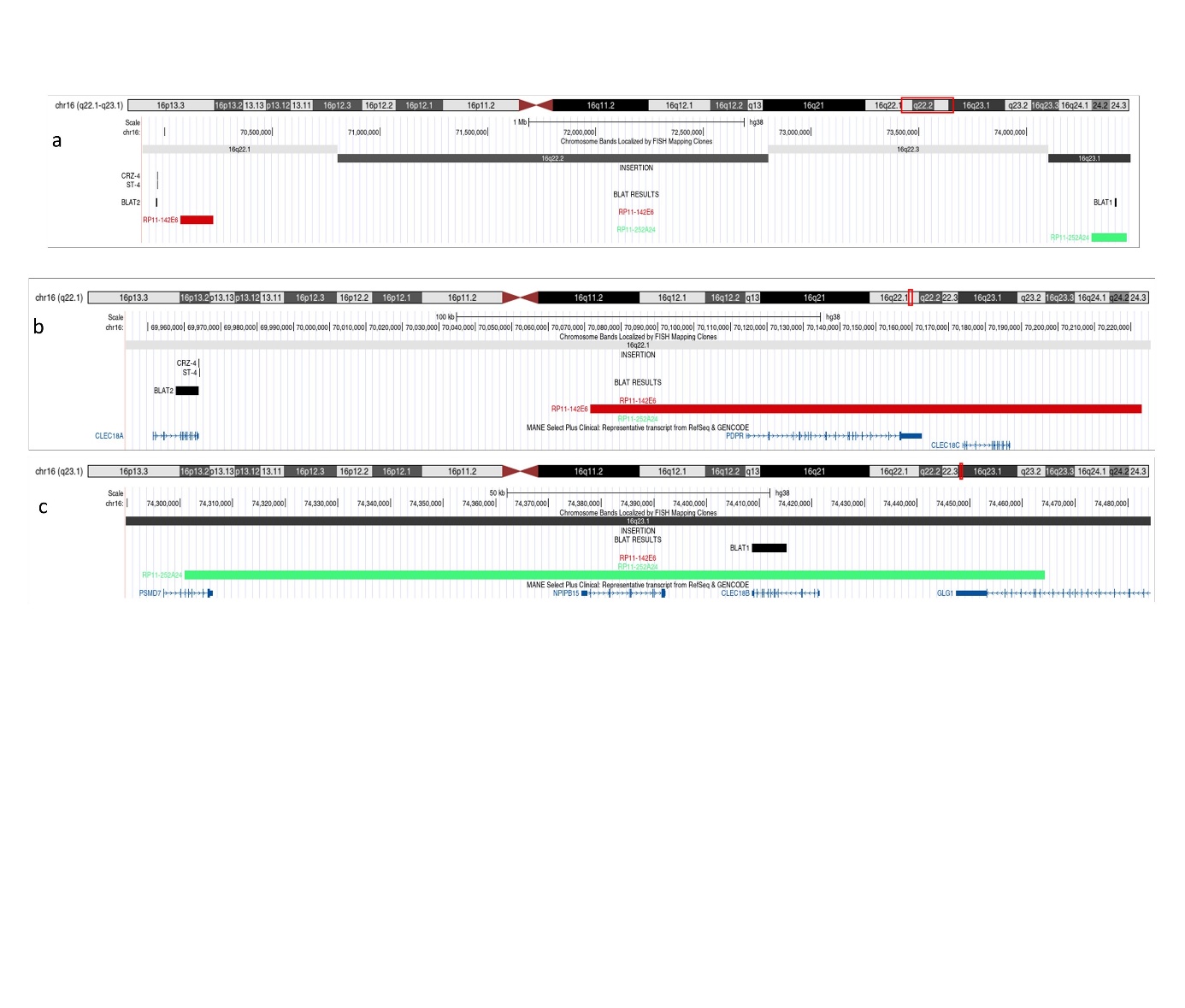

Supplement: Supplementary file 1 — Supplementary Material 1. [file 10020_2025_1415_MOESM1_ESM.docx]
